# Effects of prescribed burning regimes on soil fungal communities, functional traits, and community assembly processes in surface and subsurface peat

**DOI:** 10.1093/femsec/fiag099

**Published:** 2026-09-07

**Authors:** Shaun M Allingham, Samantha J Drake, Andrew Ramsey, Chris D Field, Felix C Nwaishi, David R Elliott

**Affiliations:** School of Biosciences, The University of Nottingham, Sutton Bonington, Loughborough LE12 5RD, United Kingdom; Nature-Based Solutions Research Centre, University of Derby, Derby DE22 1GB, United Kingdom; NERC Environmental Omics Facility, School of Biosciences, University of Sheffield, Western Bank, Sheffield S10 2TN, United Kingdom; Nature-Based Solutions Research Centre, University of Derby, Derby DE22 1GB, United Kingdom; Nature-Based Solutions Research Centre, University of Derby, Derby DE22 1GB, United Kingdom; Department of Natural Sciences, Manchester Metropolitan University, Chester Street, Manchester M1 5GD, United Kingdom; Department of Earth and Environmental Sciences, Mount Royal University, 4825 Mt Royal Gate SW, Calgary, AB T3E 6K6, Canada; Nature-Based Solutions Research Centre, University of Derby, Derby DE22 1GB, United Kingdom

**Keywords:** prescribed burning, peatlands, fungal communities, functional guilds, community assembly

## Abstract

Prescribed burning is used to manage peatlands, yet its effects on fungal communities across soil profiles remain poorly characterized. As key mediators of carbon cycling and nutrient turnover, fungi play critical roles in peatland function and resilience. We assessed fungal community dynamics under three long-term prescribed burning regimes (non-burn, long rotations every 20 years, and short rotations every 10 years) at a UK upland peatland. Observed fungal richness declined under short-rotation burning while Shannon and Simpson diversity were lower in burned plots but did not differ between long- and short-rotation treatments. Burn regime and soil depth influenced fungal composition, trophic modes, and functional guilds. Environmental drivers including pH, Ca, Mn, moss cover, and NH_4_^+^ varied with depth and were associated with shifts in community structure. Modified stochasticity ratio revealed that stochastic assembly dominated in burned plots, whereas unburned soils showed depth-dependent assembly, with deterministic processes prevailing in 0–20 cm and 40–60 cm layers. Broader niche widths in unburned plots indicated greater generalism. These findings demonstrate that prescribed burning alters community assembly and trophic modes across peat profiles. Disruption of deterministic processes and narrowing of ecological strategies under repeated prescribed burning may reduce microbial resilience, with implications for peatland sustainability.

## Teaser

Prescribed burning alters soil fungal communities even in deep peat, reshaping their ecological roles and recovery potential in ways that could impact carbon storage and long-term peatland resilience.

## Introduction

Peatlands are globally important ecosystems for carbon sequestration, regulating hydrology, and biodiversity (Holden et al. 2007, Davies et al. 2022). Although they cover only ∼3% of the Earth’s land surface they are estimated to store up to 30% of all soil carbon with current estimates of over 600 Gt making their stability vital for climate regulation (Strack et al. 2022). Peatlands, including blanket bogs, raised bogs, and upland moors, vary in their hydrology, vegetation, and nutrient availability and are actively managed to influence vegetation structure and ecosystem function. Prescribed burning is a management method utilized in peatland ecosystems throughout Europe (Hochkirch and Adorf 2007, Renard et al. 2016, Davies et al. 2022), North America (Geron and Hays 2013, Ryan et al. 2013), and the tropics (Holden et al. 2007), primarily to manage surface vegetation while minimizing combustion of the underlying peat. In the United Kingdom, burning is typically conducted on a rotational basis every 8–25 years (Noble et al. 2018). Although the official guidance discourages the prescribed burning of peat bogs in the UK (Defra 2007), the practice has historically been widespread in upland peatlands. While these peatlands are not considered classical pyrogenic ecosystems, their pyrophytic vegetation such as *Calluna vulgaris* can promote fire spread during dry periods (Beckage et al. 2011, Staver et al. 2011, Davies et al. 2016, Cardoso et al. 2018). Burning has been used to maintain vegetation mosaics and potentially enhance certain components of biodiversity (Peet et al. 2018) but can also result in substantial shifts in plant community composition, with short-rotation burning associated with increased dominance of Sphagnum moss (Lee et al. 2013, Milligan et al. 2018, Noble et al. 2018, Whitehead et al. 2021). These vegetation shifts are likely to influence fungal communities, which are tightly linked to plant hosts, litter quality, and soil chemistry. Given the central role fungi play in decomposition, nutrient cycling, and ecosystem resilience, it is essential to understand how they respond to fire-driven vegetation changes in peatland systems.

The composition and structure of plant communities exert a strong influence on fungal populations, with mutualistic associations such as mycorrhizal symbioses playing key roles in plant growth, nutrient cycling, and nutrient availability (Read et al. 2000, Allison and Treseder 2011, Žifčáková et al. 2016). In ombrotrophic peatlands, ericoid mycorrhizal fungi (ERM) are commonly associated with dominant ericaceous plants such as Calluna vulgaris (Shao et al. 2023), while arbuscular mycorrhizal fungi (AMF) are typically rare due to acidic and waterlogged conditions (Asano et al. 2021). Ectomycorrhizal fungi (EMF), commonly associated with tree hosts, are generally less abundant in ombrotrophic peatland systems due to the absence of their typical woody hosts. While EMF have been documented in surface peat layers and may contribute to plant nutrient acquisition under certain conditions, their response to prescribed burning in peatlands remains poorly understood (Thormann 2006). In peatlands, fungi perform critical functions, including the degradation of complex carbon polymers that underpin organic matter turnover under the acidic, anoxic conditions characteristic of these environments (Thormann 2006). The loss of organic matter due to wildfire or prescribed burning can significantly alter fungal community structure and shift the relative abundance of key functional guilds (Cairney and Bastias 2007, Dooley and Treseder 2012). Mycorrhizal fungi, for example, enhance plant nutrient uptake and stress tolerance, thereby supporting plant establishment, productivity, and community structure (Smith and Read 2008, Yang et al. 2016), while saprotrophic fungi are essential for the decomposition of plant litter and organic matter, processes that directly influence carbon cycling and nutrient release (Ceci et al. 2019). Given their central role in decomposition and nutrient cycling, fungi are frequently used as indicators of soil ecological recovery following disturbance. However, fungal responses are often heterogeneous, with different ecological guilds such as saprotrophs and mycorrhizal fungi exhibiting contrasting responses depending on changes in substrate availability, vegetation, and soil chemistry (Day et al. 2019, Fox et al. 2022). The majority of studies on the effects of fire on fungal communities have focused on high-intensity wildfires, where burning is more severe due to greater heat penetration into the soil profile (Cairney and Bastias 2007, Oliver et al. 2015, Reazin et al. 2016, de León et al. 2018, Whitman et al. 2019). Despite the rise of high-intensity wildfires as a result of current climate change, the majority of fires in peatlands are generally low-intensity (Turetsky et al. 2015). According to Hart et al. (2005), the effects of fire on fungal communities may differ between prescribed burns and wildfires, primarily because prescribed fires are typically lower in intensity and occur at shorter and more controlled return intervals. The resulting changes in vegetation and soil conditions can influence soil fungal communities depending on ecosystem productivity, the response of dominant plant species, and plant-fungal associations (Hart et al. 2005).

Fire influences fungal communities both directly and indirectly by altering soil conditions and vegetation structure, which are key drivers of fungal diversity and function (Certini 2005, Goldmann et al. 2016, Zhao et al. 2019). Mycorrhizal fungi are often particularly sensitive to fire and can decline following disturbance relative to other functional groups (Holden et al. 2013, Sun et al. 2015, Holden et al. 2016). As a result, ecosystem recovery following fire is closely linked to the succession of both plant and fungal communities, which often develop in concert due to strong plant-fungal interactions and feedbacks (Day et al. 2019).

Another neglected component of fungal community structure is how communities in deeper soil layers respond to prescribed burning. In peatlands, the high moisture content of deeper layers typically limits low-intensity prescribed burns to the surface, making the topsoil more affected than the subsoil (Certini 2005). Following fires, the temperature of the soil may rise due to changes in vegetation cover and then be transferred to the deeper soil via conduction, convection, and radiation; however, the effects may diminish with depth (Shakesby 2011, Pereira et al. 2018). It remains to be seen if fungal communities colonizing the deeper horizons are different to those in the upper layer or are just a subset of those from the topsoil that have reached the lower horizons due to stochastic vertical dispersion (Mujic et al. 2016, Li et al. 2020). However, most studies assessing the impacts of fire have only focused on communities in the topsoil, with a lack of data available on the effects in lower soil depths. Therefore, understanding how prescribed burning influences fungal communities across different regimes and soil depths can provide valuable insight into how peatlands are impacted by fire. A useful framework for understanding these responses is community assembly, which describes how environmental and random factors shape microbial communities.

Community assembly processes are governed by both deterministic (e.g. environmental filtering through heterogeneous and homogeneous selection) and stochastic (e.g. ecological drift, random dispersal, and diffusion limitation) mechanisms. Recent studies have demonstrated that factors such as soil properties and aboveground vegetation strongly influence the relative importance of these processes in microbial community assembly (Chen et al. 2017, Liu et al. 2021). Prescribed burning alters vegetation structure, diversity, and biomass which may create heterogeneous conditions in the soil environment. These disturbances are expected to weaken deterministic selection pressures leading to a more stochastic community due to colonization and ecological drift (Zhang et al. 2022, Wang et al. 2024). By contrast, an unburned area provides more stable environmental conditions where soil properties and plant-driven inputs promote strong environmental filtering, favouring deterministic processes. Thus, investigating fungal assembly under prescribed burning conditions is essential to deepen our understanding of fungal diversity and adaptive strategies in response to environmental disturbances (Sawada et al. 2023).

In this study, we used long-term experimental burning plots at Moor House National Nature Reserve in northern England. The site includes prescribed burning treatments with established rotation intervals, providing a well-characterized framework to examine how burn frequency influences fungal communities in peatland soils. The study aimed to address two main questions: (Q1) Do prescribed burns affect soil fungal communities throughout the soil profile? (Q2) Do these responses depend on different burning regimes (non-burn, long-rotation, or short-rotation)? To address these questions, we tested the following hypotheses: (H1) Prescribed burning will significantly alter fungal community richness and composition with short-rotation burns exhibiting the strongest effects due to more repeated prescribed burning; (H2) Stochastic processes will be the dominant driver of fungal community assembly in burned plots due to increased disturbance and spatial heterogeneity, whereas deterministic processes will prevail in unburned plots due to environmental filtering; (H3) Prescribed burning will favour saprotrophic fungi adapted to resource limitation and disturbance, whereas symbiotrophic fungi will be more abundant in non-burned plots due to stable plant-fungal interactions. These findings will provide insights into how rotational burning shapes the structure and ecological strategies of soil fungal communities with implications for belowground resilience, carbon storage potential, and the long-term sustainability of peatland fire management strategies.

## Materials and methods

### Study site, experimental design, and soil physicochemical measurements

Details of the study site, experimental design, and soil physicochemical measurements used in this study were previously described by Allingham et al. (2024). Briefly, the experiment was carried out at the Hard Hill long-term burning experiment (54°43′N 2°23′W) in July 2020. The long-term programme was established in 1954 on an area of blanket peatland at Moor House-Upper Teesdale National Nature Reserve (NNR) in the North Pennines, UK.

Soil sampling was conducted in July 2020. The experiment is divided into four blocks, each containing six 30 m × 30 m plots arranged in a randomized block split-plot design combining two treatments (fenced and grazed) with three prescribed burning regimes: no burn since 1954 (non-burn), burning at 20-year intervals (long-rotation, intermediate burn), and burning at 10-year intervals (short-rotation, most intensively burned). The short-rotation plots have been burned seven times and the long-rotation plots four times since the establishment of the experimental plots in 1954 and have been used to monitor how plant communities respond to prescribed burning (e.g. Lee et al. 2013, Milligan et al. 2018, Noble et al. 2018, 2019). A limitation of this experimental design is that treatments vary not only in fire frequency but also in the interval between burns. For instance, the non-burn plots have not been burned since 1954, whereas the short-rotation and long-rotation treatments were last burned in 2017 (Clutterbuck et al. 2020), meaning that time since fire is consistent between the two burn regimes. As such, treatment effects should be interpreted as reflecting overall fire regime rather than burn frequency alone, allowing us to assess how these historical regimes influence fungal communities.

In this study, soil sampling was conducted only within the fenced plots to avoid potential grazing effects and to focus specifically on the influence of prescribed burning. Within each burn treatment in each block, three 1 m × 1 m quadrats were randomly established within the fenced plot, giving three quadrats per treatment per block and a total of twelve quadrats per burn treatment across the four blocks (*n* = 36). Within each quadrat, % vegetation cover was estimated. Plants were classified into morphological groups including heather, graminoids, Sphagnum moss, other non-Sphagnum mosses, and other vascular plants.

Next, five soil cores were collected vertically using a 10 mm diameter Haglöf Soiltax soil sampler, with one core taken from each corner of the quadrat and one from the centre at each of three depth intervals of 0–20 cm, 20–40 cm, and 40–60 cm. The five subsamples collected from each quadrat at each depth were pooled to create a single composite sample for chemical and microbial analyses. A total of 12 samples per treatment and per depth were collected (a total of 108 samples). Soil samples for microbial analysis were placed into sterile Whirl-Pak® bags immediately after collection, stored in a cooler with ice packs in the field, and transferred to a −20°C freezer within 6 h of sampling. The soil characteristics of sampling sites are shown in Table S1.

### DNA extraction

DNA was extracted using the DNeasy® PowerSoil® kit (Qiagen, Manchester, UK). Due to the low bulk density of peat soil, 0.10 g of freeze-dried soil was weighed instead of the normally recommended 0.25 g wet soil suggested by the kit instructions. This method adjustment was done after consultation with the manufacturer. The soil was homogenized and rewetted with 150 µl of nuclease-free water. The rest of the manufacturer’s instructions were followed, with the addition of a 30 min incubation period at 65°C following the addition of the C1 lysis buffer and 10 min of vortexing. The extracted DNA was quantified using a Qubit4 Fluorometer (Invitrogen, UK) and stored at −20°C for subsequent analysis.

### PCR amplification and next generation sequencing

Extracted DNA was used as a template for next generation sequencing using the two step PCR approach. Fungal communities were specifically targeted by amplifying the ITS regions in the nuclear ribosomal repeat using the primer pair ITS1F (5′-CTTGGTCATTTAGAGGAAGTAA-3′) and ITS2R (5′-GCTGCGTTCTTCATCGATGC-3′) (Bokulich and Mills 2013, Hugerth et al. 2014). PCR reactions were performed using a 20 µl mixture containing 10 µl 2x Qiagen Multiplex PCR master mix, 2 µl forward primer, 2 µl reverse primer, 5 µl of RNase/DNase-free water, and 1 µl of DNA template. The first PCR was carried out in a thermocycler PCR system (MJ Research ptc-225 peltier thermal cycler) using the following program: 3 min of denaturation at 95°C, 35 cycles of 30 s at 95°C, 30 s for annealing at 53°C, and 45 s for elongation at 72°C, and a final extension at 72°C for 10 min (Pauvert et al. 2019). The presence of a PCR product was confirmed using 1% agarose gel electrophoresis. PCR products were then cleaned using Agencourt AMPure XP magnetic beads (Beckman Coulter, Indianapolis, USA). Each sample was subjected to a second PCR with barcoded Fi5 and Ri7 identifier sequences. The second PCR mixtures contained 1 µl of Fi5 primer, 1 µl Ri7 primer, 8 µl of product from the first PCR, and 10 µl of Qiagen multiplex master mix, a total volume of 20 µl. The following program was used: 95°C for 15 min followed by 12 cycles of 98°C for 10 s, 65°C for 30 s, and 72°C for 30 s. Following the second PCR, a FLUOstar Optima (Promega) was used to measure the DNA concentration using 2 µl of product from each reaction. Based on these results samples were standardized to equal concentrations, pooled into groups of 12 and cleaned using AMPure XP beads (Beckman Coulter, Indianapolis, USA). The Illumina-tagged DNA concentration of each pool was determined using the KAPA Library Quantification Kit on an Applied Biosystems QuantStudio 12 K, and DNA fragment size was determined using an Agilent 2100 Bioanalyzer (Agilent Technologies Ltd., Stockport, UK). The KAPA Library Quantification Kit and a Qubit 3.0 with the dsDNA HS test (Invitrogen, UK) were used to quantify the final pools. Libraries were sequenced on an Illumina MiSeq platform at 2 × 250 bp paired-end sequencing (Magoč and Salzberg 2011) at the NERC Environmental Omics Facility within the Centre for Genomic Research, University of Liverpool. The raw reads were deposited into the National Center of Biotechnology Information (NCBI) Sequence Read Archive (SRA) database under the BioProject accession number PRJNA1002628.

### Bioinformatics

Bioinformatics analysis was conducted using QIIME2 v2019.7 (Bolyen et al. 2019). First, primer sequences were removed using cutadapt v1.9.1 (Martin 2011). The conserved flanking regions of the ITS1 reads were trimmed using ITSxpress (v 1.8.0) as recommended for amplicon sequencing (Rivers et al. 2018). DADA2 was used to filter, dereplicate, merge paired-end reads, and remove chimeras (Callahan et al. 2016). The q2-dada2 plugin uses nucleotide quality scores to infer amplicon sequence variants (ASVs), which aim to recover biological sequence variation while reducing artefacts introduced by sequencing errors. The fungal ITS region is highly variable in length and thus was not length trimmed but was quality filtered. Parameters were set with a maxEE score of 2 and truncQ score of 10. The UNITE fungal ITS sequence database (version 8.3) was used as a reference database assigning ASVs to a taxonomic classification (Kõljalg et al. 2013). The q2-feature-classifier in QIIME2 (https://github.com/qiime2/q2-feature-classifier) was used to assign taxonomy using a confidence threshold of >0.70. Singletons were discarded as recommended by Lindahl et al. (2013). Rarefaction curves were generated using the R package ‘*ampvis2*’ (Andersen et al. 2018). Samples were rarefied to the minimum read depth for normalization (Fig. S1), and alpha diversity metrics including observed richness, Shannon index, and Simpson index were calculated using the R package phyloseq (McMurdie and Holmes 2013). Samples that had very low reads (<1000) were considered insufficient for analysis and were removed.

Functional guild analysis was performed using FUNGuild (Nguyen et al. 2016) located at https://github.com/UMNFuN/FUNGuild. Using the FUNGuild data, the ‘overall’ fungal community was divided into trophic modes: pathotroph, saprotroph, symbiotroph, and multiple trophic modes. The ‘multiple’ category included ASVs assigned to more than one trophic mode (e.g. pathotroph–saprotroph, symbiotroph–pathotroph–saprotroph), reflecting taxa with overlapping or flexible ecological strategies. Only taxa with ‘probable’ or ‘highly probable’ confidence scores were retained for analysis. Fungal sequences that were not assigned a trophic mode were labelled ‘unknown’.

### Statistical analysis

All statistical analyses were conducted in R version 4.0.2 (R Core Team 2020). The effects of burn regime and soil depth on fungal alpha diversity (Observed richness, Shannon, Simpson), fungal taxonomic composition, and fungal trophic modes were analyzed using linear mixed-effects models fitted by restricted maximum likelihood (REML), with burn regime and soil depth as fixed effects, block as a random effect, and quadrat identity as an additional random effect to account for repeated depth samples within quadrats. Type III analysis of variance (ANOVA) with Satterthwaite’s approximation was used to assess the significance of fixed effects. Where significant effects were detected, pairwise comparisons were performed using Tukey-adjusted estimated marginal means. Relative abundance data for fungal taxa and trophic modes were treated as compositional and therefore transformed using the centred log-ratio (CLR) transformation prior to analysis. Statistical tests were conducted on CLR-transformed data, while relative abundances are presented for ease of ecological interpretation.

For analysis of community composition (β-diversity), the ASV table was normalized by converting read counts to relative abundances (total sum scaling, TSS) using the R package *microbiomeSeq* (Ssekagiri et al. 2017) as this method is efficient at standardizing read depths while preserving relative abundances (McKnight et al. 2019). Bray–Curtis dissimilarity matrices were calculated using the ‘vegan’ package (Oksanen 2015) and visualized using principal coordinates analysis (PCoA; Gower 1966). Permutational multivariate analysis of variance (PERMANOVA) was conducted using the adonis2 function with 999 permutations in *Vegan*, including burn regime, soil depth, and their interaction as fixed effects, and block included as a strata term to account for the experimental design. Prior to ordination, ASVs with a total relative abundance of less than 0.001 were excluded to minimize the influence of rare taxa (Legendre and Gallagher 2001).

The “NST” package in R was used to calculate MST in order to understand the microbial community assembly. Deterministic processes may be more significant in community assembly when MST is less than 50%, while stochastic processes may be more significant when MST is greater than 50% (Zhou et al. 2014, Ning et al. 2019). A Sloan Neutral Community Model (NCM) was applied using the R package minpack.lm to assess the relative contributions of stochastic processes such as ecological drift and dispersal limitation in structuring fungal communities (Sloan et al. 2006). The model relates the mean relative abundance of taxa to their frequency of occurrence across samples assuming community dynamics are governed by neutral stochasticity. The migration parameter (m) was estimated using nonlinear least-squares regression. Confidence intervals for m were calculated using 1000 bootstrap replicates. Model fit was evaluated using *R^2^*, and observed frequencies were compared with predicted frequencies using Wilson confidence intervals. Deviations from the model’s predictions suggest the influence of non-neutral processes.

The mean habitat niche width for each burn regime and depth in fungal communities was estimated using the ‘spaa’ package in R based on the Levins' niche width approach described by Zhang et al. (2018). ASVs were classified as neutral taxa when their observed niche breadth was within the 95% CI range, and as generalists (>95%) or specialists (<95%) when their observed niche breadth was greater than the upper 95% CI or less than the lower 95% CI (Wu et al. 2017, Salazar 2020). The “EcolUtils” package in R was used to calculate the relative abundance of generalist, neutral, and specialist species of fungi (Salazar 2020).

Modified stochasticity ratio (MST) and Levins’ niche width were analyzed using linear mixed-effects models with burn regime and soil depth as fixed effects, block as a random effect and quadrat identity as an additional random effect to account for repeated depth samples within quadrats. Because MST is bounded between 0 and 100, MST values were logit-transformed prior to analysis to meet model assumptions. Levins’ niche width values were log-transformed prior to analysis to improve normality and homoscedasticity of residuals. Significance of fixed effects was assessed using Type III ANOVA, and pairwise comparisons were performed using Tukey-adjusted estimated marginal means.

After standardizing the ASV abundance matrix using Hellinger transformation, a redundancy analysis (RDA) was performed on the topsoil (0–20 cm) and subsoils (20–40 cm and 40–60 cm) to visualize differences in fungal community composition between treatments and to test the importance of environmental factors. The ‘best’ explanatory environmental variables were chosen through forward selection using the ordistep function (Blanchet et al. 2008). Environmental variables confirmed by analysis of variance were retained for the final RDA. The variation inflation factor (VIF) was used to check non-collinearity among the explanatory variables (VIF < 10), as recommended by Montgomery and Peck (1992).

Indicator species analysis was performed using the function multipatt in the R package indicspecies to identify taxa significantly associated with particular burn regimes or depths, representing those that are ecologically indicative of specific environmental conditions. The group-equalized IndVal statistic (IndVal.g) was used, and statistical significance was assessed using permutation tests (9999 permutations). Associations with *P* < 0.05 were considered significant.

## Results

### General characteristics of fungal communities across burn regimes and depth

**Figure 1:**
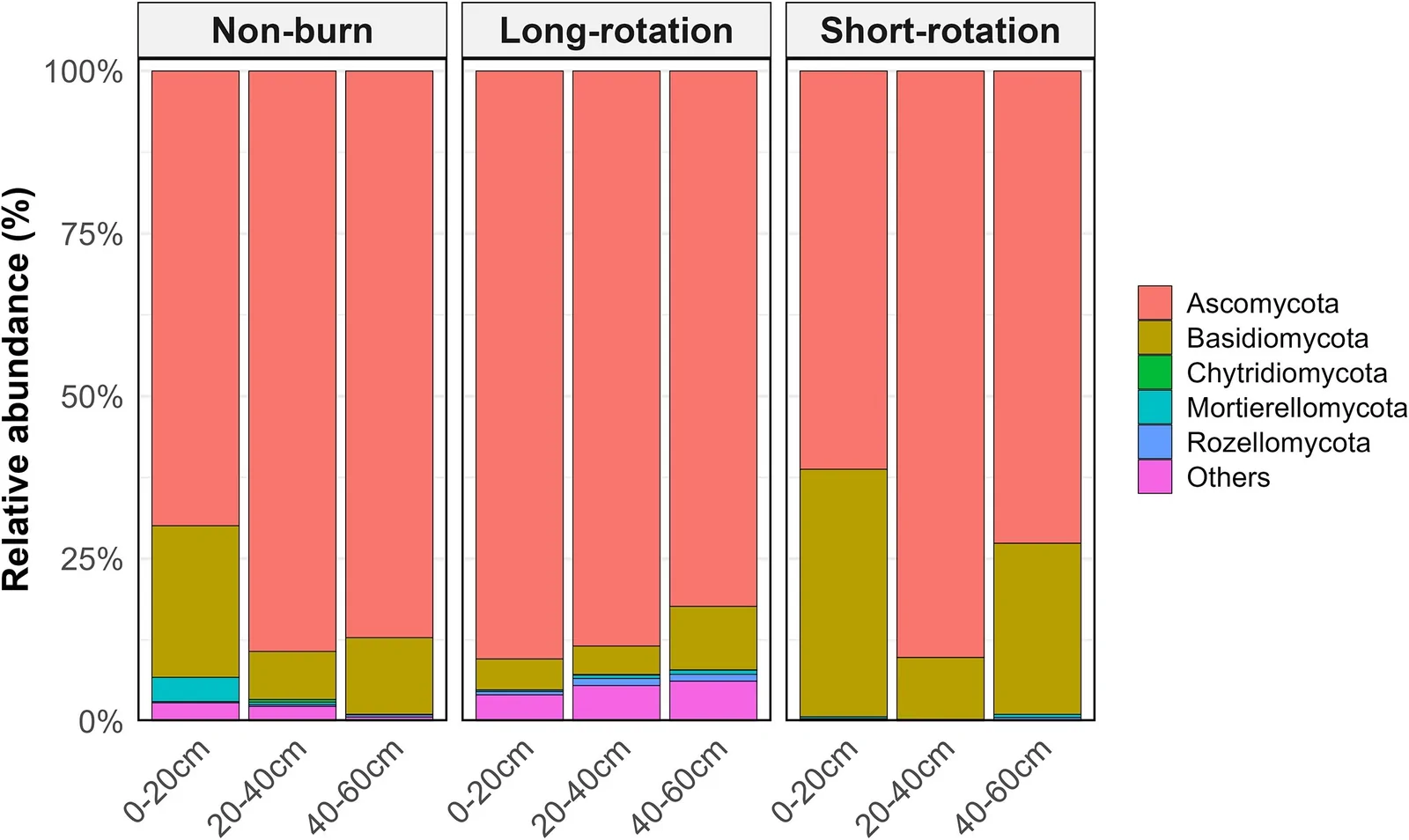
Relative abundance of the top five fungal phyla across three different soil depths under three prescribed burn regimes: Non-burn (unburned since 1954), long-rotation (20-year interval), and short-rotation (10-year interval). Sample sizes: Non-burn 0–20 cm (*n* = 12), non-burn 20–40 cm (*n* = 12), non-burn 40–60 cm (*n* = 9), long-rotation 0–20 cm (*n* = 12), long-rotation 20–40 cm (*n* = 11), long-rotation 40–60 cm (*n* = 12), short-rotation 0–20 cm (*n* = 12), short-rotation 20–40 cm (*n* = 12), short-rotation 40–60 cm (*n* = 12).

Eight fungal phyla were detected across all samples. The phylum Ascomycota showed the highest relative abundance across all samples averaging 81%, with the highest relative abundance in the topsoil of plots under a long-rotation regime (90%) and the lowest relative abundance in plots under a short-rotation regime (61%). The relative abundance of Ascomycota stayed relatively constant across soil depth (Fig. 1). The relative abundance of Basidiomycota averaged 16% across all samples being highest in the topsoil in plots under a short-rotation regime (38%) and lowest in long-rotation plots, at 4% in both 0–20 and 20–40 cm layers. The phylum Mortierellomycota was higher in the topsoil of the non-burn control plots compared to plots under long-rotation and short-rotation burn regimes (Fig. 1).

**Figure 2:**
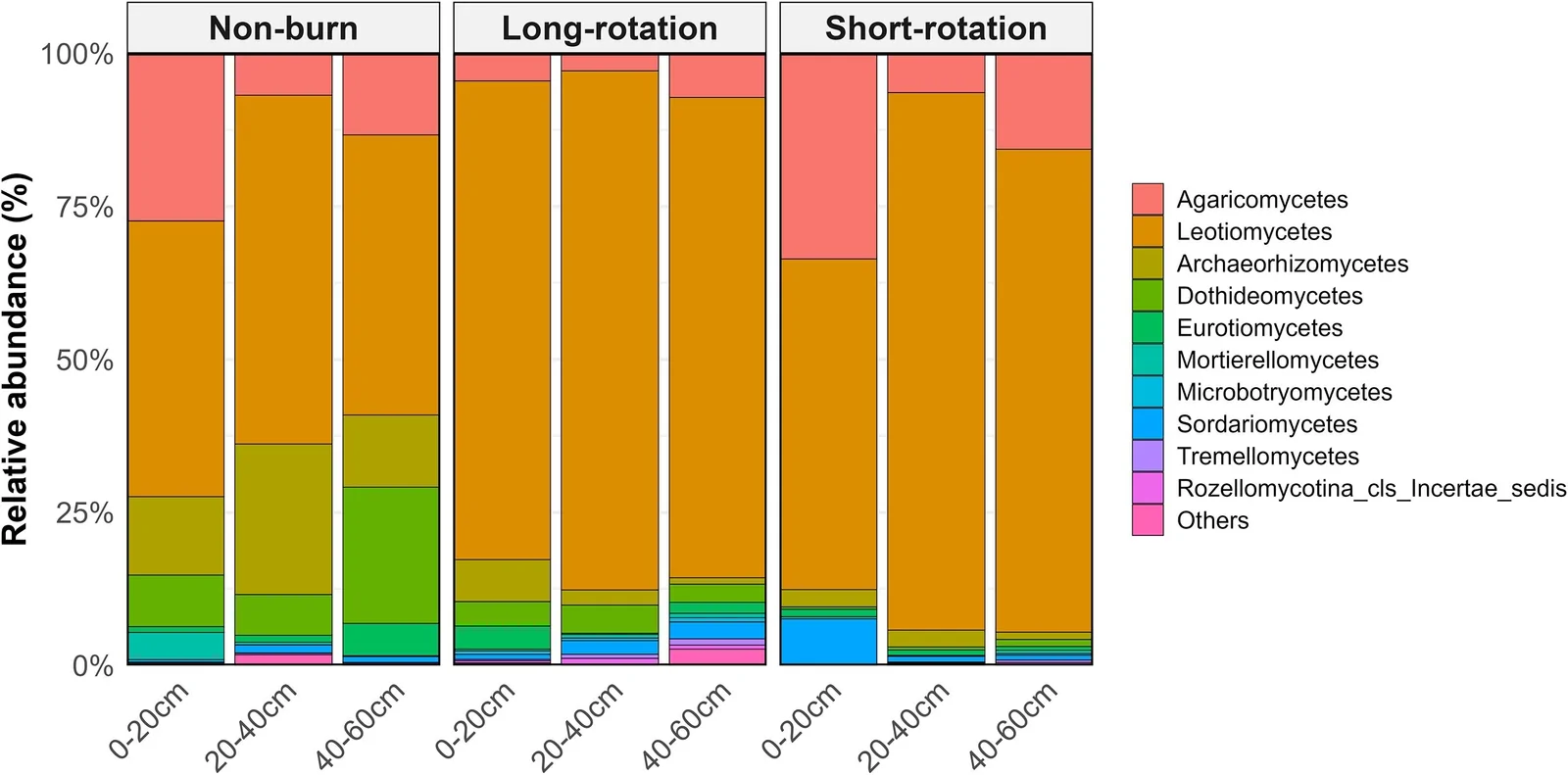
Relative abundance of the top ten fungal classes across three different soil depths under three prescribed burn regimes: Non-burn (unburned since 1954), long-rotation (20-year interval), and short-rotation (10-year interval). Sample sizes: Non-burn 0–20 cm (*n* = 12), non-burn 20–40 cm (*n* = 12), non-burn 40–60 cm (*n* = 9), long-rotation 0–20 cm (*n* = 12), long-rotation 20–40 cm (*n* = 11), long-rotation 40–60 cm (*n* = 12), short-rotation 0–20 cm (*n* = 12), short-rotation 20–40 cm (*n* = 12), short-rotation 40–60 cm (*n* = 12).

Across all samples, fungal communities were dominated by the class Leotiomycetes comprising 61.2% of total reads. Other abundant classes included Agaricomycetes (11.5%) and Archaeorhizomycetes (7%). All other identified classes were present at relative abundances below 5%, with unidentified taxa accounting for 8.13% of total reads (Fig. 2). The relative abundance of Leotiomycetes was lowest in the non-burn control across all three depth profiles (Fig. 2). The abundance of Agaricomycetes generally decreased with depth except for under a long-rotation regime (*F*(4, 95) = 3.96, *P* = 0.02) and was significantly different across burn regimes (*F*(2, 95) = 26.21, *P* < 0.001) being highest in plots under a short-rotation regime (Table S2). However, the abundance of Archaeorhizomycetes was greater in non-burn soils than in burned soils (*F*(2, 30.40) = 8.93, *P* < 0.001). Members of the classes Dothideomycetes, Eurotiomycetes, Microbotryomycetes, Mortierellomycetes, Rozellomycotina_cls_Incertae_sedis, Sordariomycetes, and Tremellomycetes were found at low relative abundance (Fig. 2). The classes Agaricomycetes, Archaeorhizomycetes, Dothideomycetes, and Leotiomycetes were all significantly affected by burn regime, while Eurotiomycetes showed a significant burn regime × soil depth interaction (*P* < 0.05) (Table S2). In the topsoil, several genera showed lower relative abundance in burned plots than in non-burn plots. Under long-rotation burning, Serendipita declined from 18.7% to 2.6%, Archaeorhizomyces from 10.9% to 6.1%, and Mortierella from 3.8% to 0.3%, while Venturia was absent. Under short-rotation burning, Archaeorhizomyces declined to 2.6%, Mortierella to 0.3%, Penidiella to 0.01%, and Protoventuria was absent, while Venturia was again absent (Fig. S2).

### Fungal community diversity and community composition across burn regimes and depth

**Figure 3:**
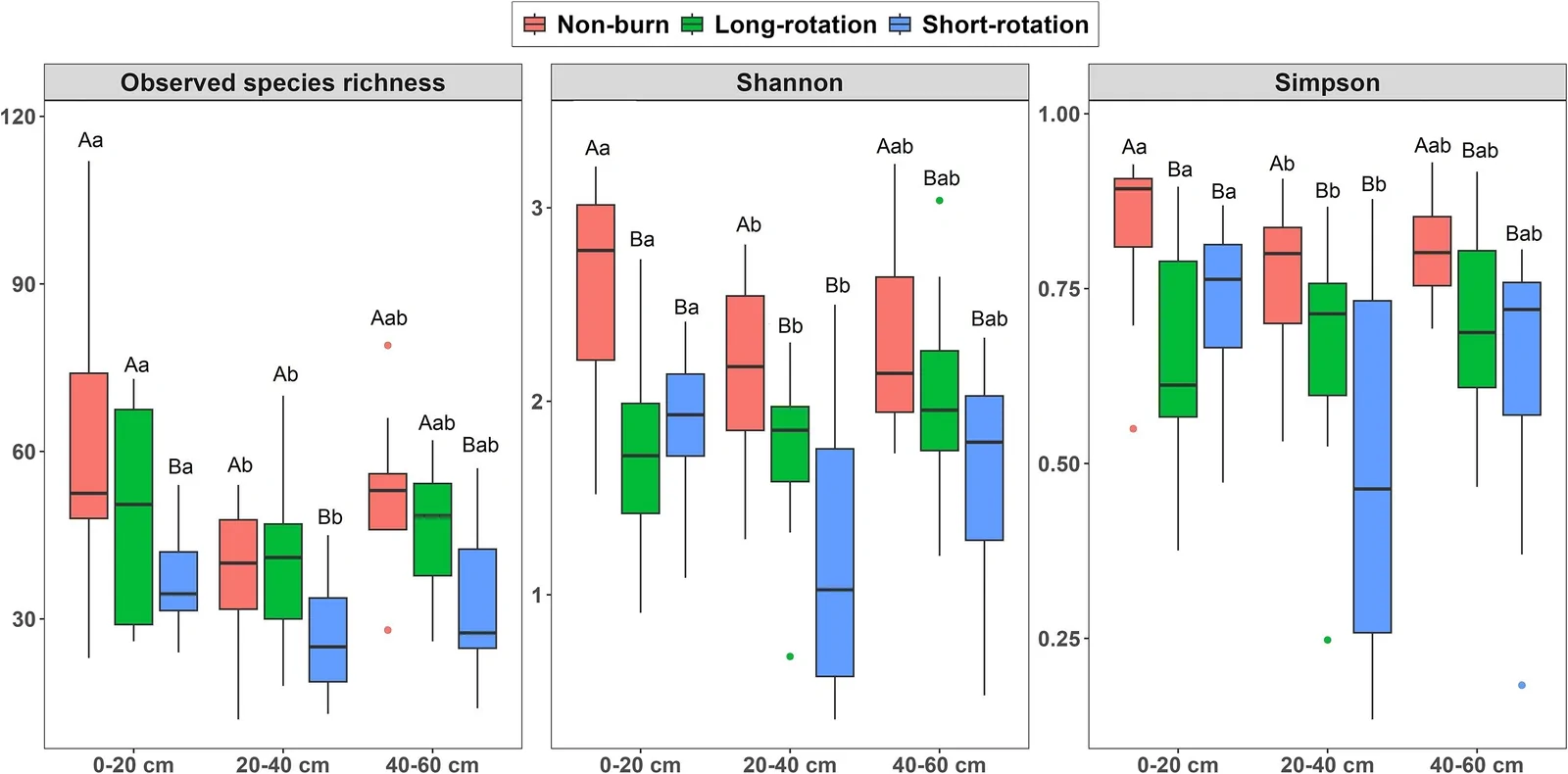
Alpha diversity indices of fungal communities across three different soil depths under three prescribed burn regimes. Observed richness (A), Shannon index (B), and Simpson index (C). Statistical differences were assessed using linear mixed-effects models, with significance determined from Type III ANOVA and Tukey-adjusted pairwise comparisons (*P* < 0.05). Letters represent significant main effects: uppercase letters indicate significant differences among burn treatments averaged across soil depths, and lowercase letters indicate significant differences among soil depths averaged across burn treatments. Prescribed burn regimes: Non-burn (unburned since 1954), long-rotation (20-year interval), and short-rotation (10-year interval). Sample sizes: Non-burn 0–20 cm (*n* = 12), non-burn 20–40 cm (*n* = 12), non-burn 40–60 cm (*n* = 9), long-rotation 0–20 cm (*n* = 12), long-rotation 20–40 cm (*n* = 11), long-rotation 40–60 cm (*n* = 12), short-rotation 0–20 cm (*n* = 12), short-rotation 20–40 cm (*n* = 12), short-rotation 40–60 cm (*n* = 12).

All three alpha diversity metrics (observed richness, Shannon, and Simpson) differed significantly among prescribed burn regimes and across soil depths. However, no significant interaction between burn regime and depth was found for any of the metrics (Fig. 3; Table S3). Observed richness was significantly different between burn regime (*F*(2, 33.35) = 12.04, *P* <0.001) and depth (*F*(2, 64.61) = 7.59, *P* = 0.001) but no interaction (*F*(4, 64.50) = 0.61, *P* = 0.69). Observed richness was highest in the topsoil of non-burned plots and long rotation plots and declined with depth. Similarly, Shannon diversity was significantly different between burn regimes (*F*(2, 32.57) = 15.73, *P* <0.001) and depth (*F*(2, 64.35) = 4.38, *P* = 0.01), with no significant interaction (*F*(4, 64.13) = 1.66, *P* = 0.17). Simpson diversity followed the same pattern, showing burn regime had a significant effect (*F*(2, 32.67) = 9.58, *P* < 0.001) as well as depth (*F*(2, 64.70) = 4.26, *P* = 0.01) but no significant interaction (*F*(4, 64.52) = 1.89, *P* = 0.12). Both Shannon and Simpson diversity were highest in non-burn plots and lower in long-rotation and short-rotation plots, which did not differ significantly from each other (Fig. 3; Table S3).

**Figure 4:**
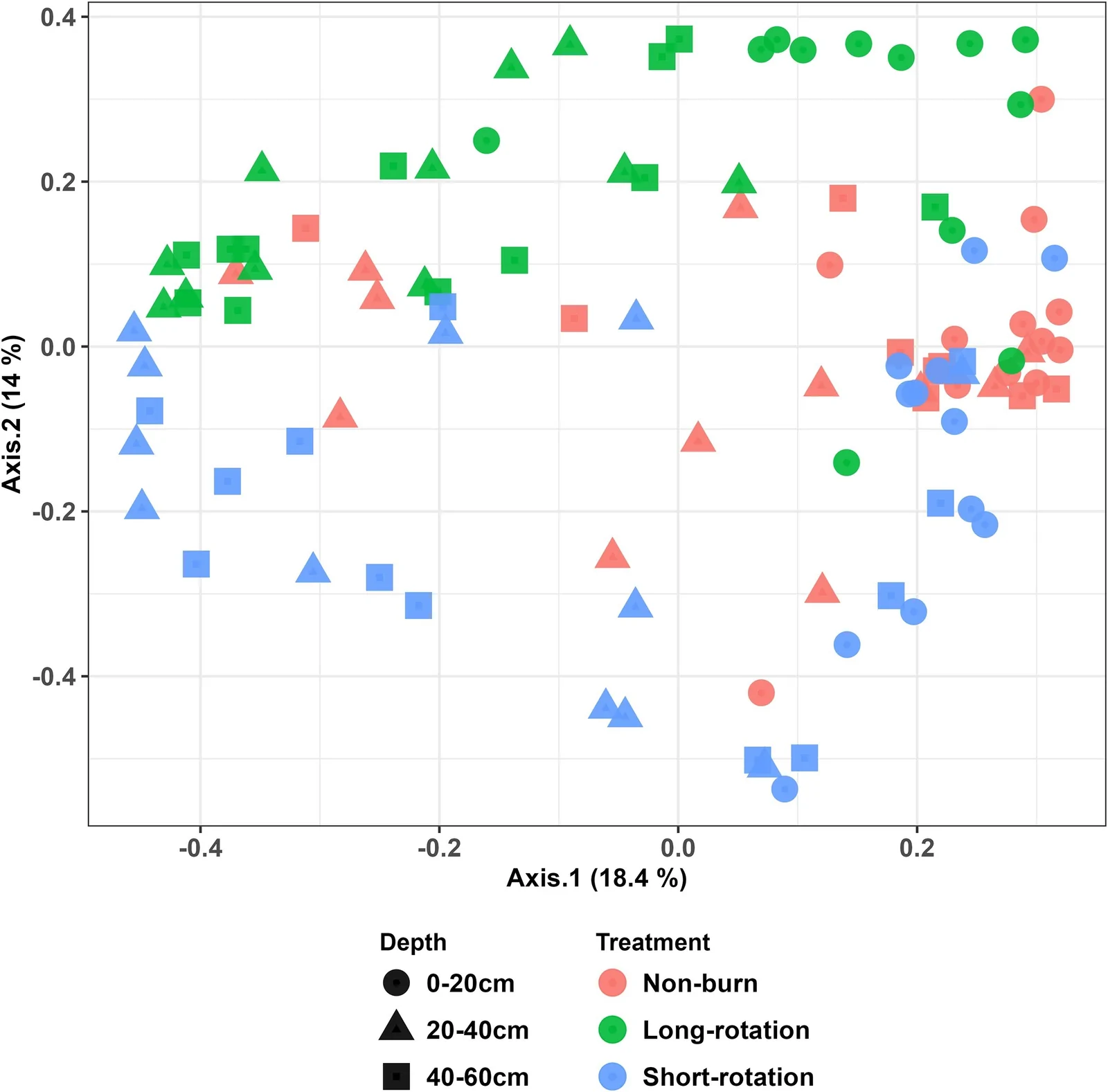
Principal coordinate analysis based on the Bray–Curtis dissimilarity of fungal community structure for soil samples collected across three different soil depths under three prescribed burn regimes: Non-burn (unburned since 1954), long-rotation (20-year interval), and short-rotation (10-year interval). Shapes represent soil depth: circles = 0–20 cm, triangles = 20–40 cm, squares = 40–60 cm. Sample sizes: Non-burn 0–20 cm (*n* = 12), non-burn 20–40 cm (*n* = 12), non-burn 40–60 cm (*n* = 9), long-rotation 0–20 cm (*n* = 12), long-rotation 20–40 cm (*n* = 11), long-rotation 40–60 cm (*n* = 12), short-rotation 0–20 cm (*n* = 12), short-rotation 20–40 cm (*n* = 12), short-rotation 40–60 cm (*n* = 12).

Using the Bray–Curtis dissimilarity, principal coordinate analysis (PCoA) was conducted to illustrate the variance of fungal community structure along the soil depth gradients in different burn regimes. Overall, the PCoA (Fig. 4) showed that beta diversity of fungal communities was significantly different between burn regimes (PERMANOVA, *F*(2,95) = 8.62, *R^2^* = 0.135, *P* = 0.001) and soil depths (*F*(2,95) = 5.27, *R^2^* = 0.083, *P* = 0.001), while the interaction between burn treatment and depth was not significant (*F*(4,95) = 1.24, *R^2^* = 0.039, *P* = 0.08). Overall, samples showed clear separation according to depth and burn regime while samples under the same treatment tended to cluster closer together (Fig. 4).

### Community assembly processes

**Figure 5:**
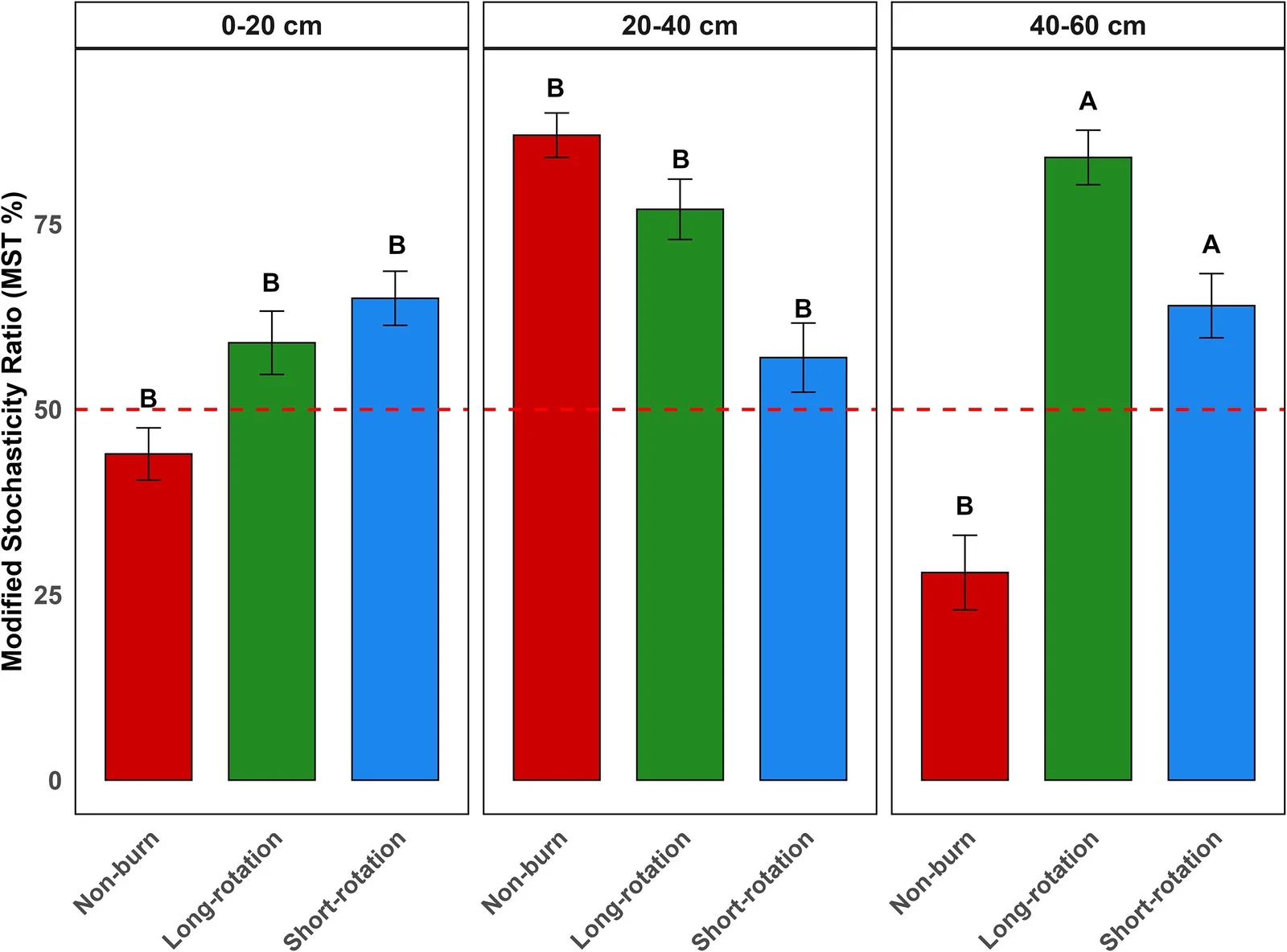
Modified stochasticity ratio (MST) of fungal communities across three different soil depths under three prescribed burn regimes: Non-burn (unburned since 1954), long-rotation (20-year interval), and short-rotation (10-year interval). The dashed line at an MST value of 50 indicates the threshold separating stochastic (MST > 50) from deterministic (MST < 50) assembly processes. Statistical differences were assessed using linear mixed-effects models applied to logit-transformed data. Letters indicate significant differences among burn treatments within each soil depth based on Tukey-adjusted pairwise comparisons (*P* < 0.05). Values are presented as raw MST percentages for interpretation. Differences are only interpreted within individual soil depths due to a significant treatment × depth interaction. Sample sizes: Non-burn 0–20 cm (*n* = 12), non-burn 20–40 cm (*n* = 12), non-burn 40–60 cm (*n* = 9), long-rotation 0–20 cm (*n* = 12), long-rotation 20–40 cm (*n* = 11), long-rotation 40–60 cm (*n* = 12), short-rotation 0–20 cm (*n* = 12), short-rotation 20–40 cm (*n* = 12), short-rotation 40–60 cm (*n* = 12).

Modified stochasticity ratio (MST) was significantly influenced by burn treatment (*F*(2, 55.84) = 8.37, *P* < 0.001), soil depth (*F*(2, 504.17) = 9.97, *P* < 0.001), and their interaction (*F*(4, 503.38) = 5.45, *P* < 0.001), indicating that the effect of burning on community assembly processes varied across the soil profile. At 0–20 cm and 40–60 cm, MST values in non-burn plots were below 50%, indicating more deterministic assembly, whereas burned treatments showed higher MST values, suggesting a shift towards more stochastic processes (Fig. 5; Table S4). In contrast, at 20–40 cm, MST values were more similar among burn treatments.

The Sloan Neutral model (NCM) explained community assembly in some treatments but showed poor fit (- *R^2^*) in others. Non-burn plots at 0–20 cm and 20–40 cm exhibited moderate fits (*R^2^* = 0.281 and 0.239) and low migration rates (m = 0.008 and m = 0.00466). However, all other treatments, including deeper soil layers and long and short-rotation burn regimes had negative *R^2^* values indicating a poor alignment with NCM (Fig. S3). The metacommunity size × immigration between communities (Nm-value) ranged widely from 105.18 to 559.05 across treatments (Fig. S3).

**Figure 6:**
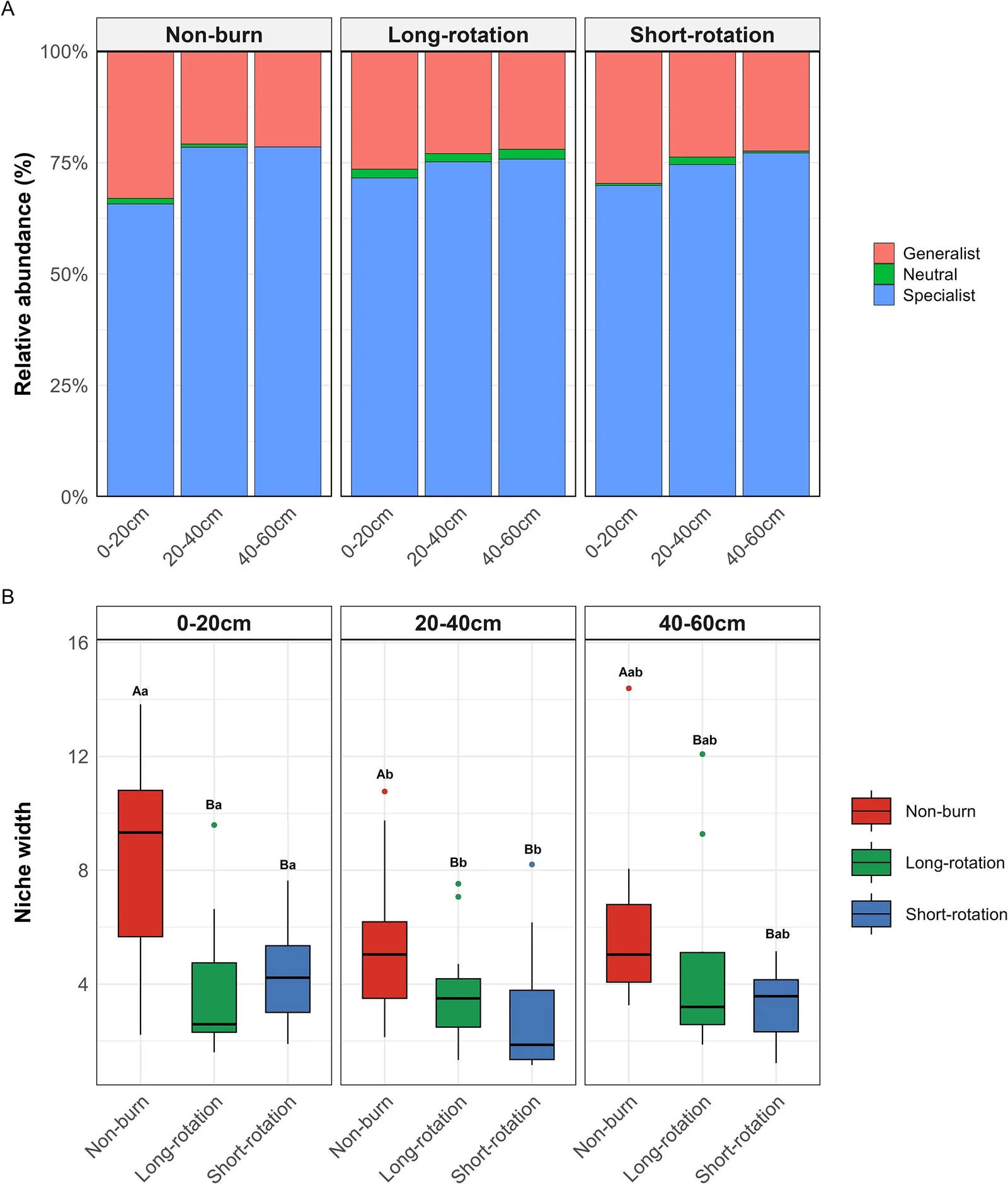
(A) Relative abundance of fungal taxa classified as generalists, neutrals, and specialists across three different soil depths under three prescribed burn regimes: Non-burn (unburned since 1954), long-rotation (20-year interval), and short-rotation (10-year interval). (B) Levins’ niche width across three soil depths under three prescribed burn regimes. Statistical differences were assessed using linear mixed-effects models applied to log-transformed data, with significance determined from Type III ANOVA and Tukey-adjusted pairwise comparisons (*P* < 0.05). Values are presented as raw values for interpretation. Uppercase letters indicate significant differences among burn treatments, and lowercase letters indicate significant differences among soil depths. Sample sizes: Non-burn 0–20 cm (*n* = 12), non-burn 20–40 cm (*n* = 12), non-burn 40–60 cm (*n* = 9), long-rotation 0–20 cm (*n* = 12), long-rotation 20–40 cm (*n* = 11), long-rotation 40–60 cm (*n* = 12), short-rotation 0–20 cm (*n* = 12), short-rotation 20–40 cm (*n* = 12), short-rotation 40–60 cm (*n* = 12).

EcolUtils classification revealed that specialist fungi dominated all treatments and depths (65%–78%). However, the relative abundance of generalists was highest in the topsoil (0–20 cm) of non-burn (33%) and short-rotation plots (30%), with slightly lower proportions in long rotation plots (26%) (Fig. 6A). Levins’ niche width was significantly affected by burn treatment (*F*(2, 31.74) = 12.44, *P* < 0.001) being higher in non-burn plots compared to burned treatments. A significant effect of soil depth was also detected (*F*(2, 63.67) = 3.41, *P* = 0.03). No significant treatment × depth interaction was observed (*F*(4, 63.48) = 1.03, *P* = 0.40) (Fig. 6B).

### Environmental factors influencing soil fungal communities

**Figure 7:**
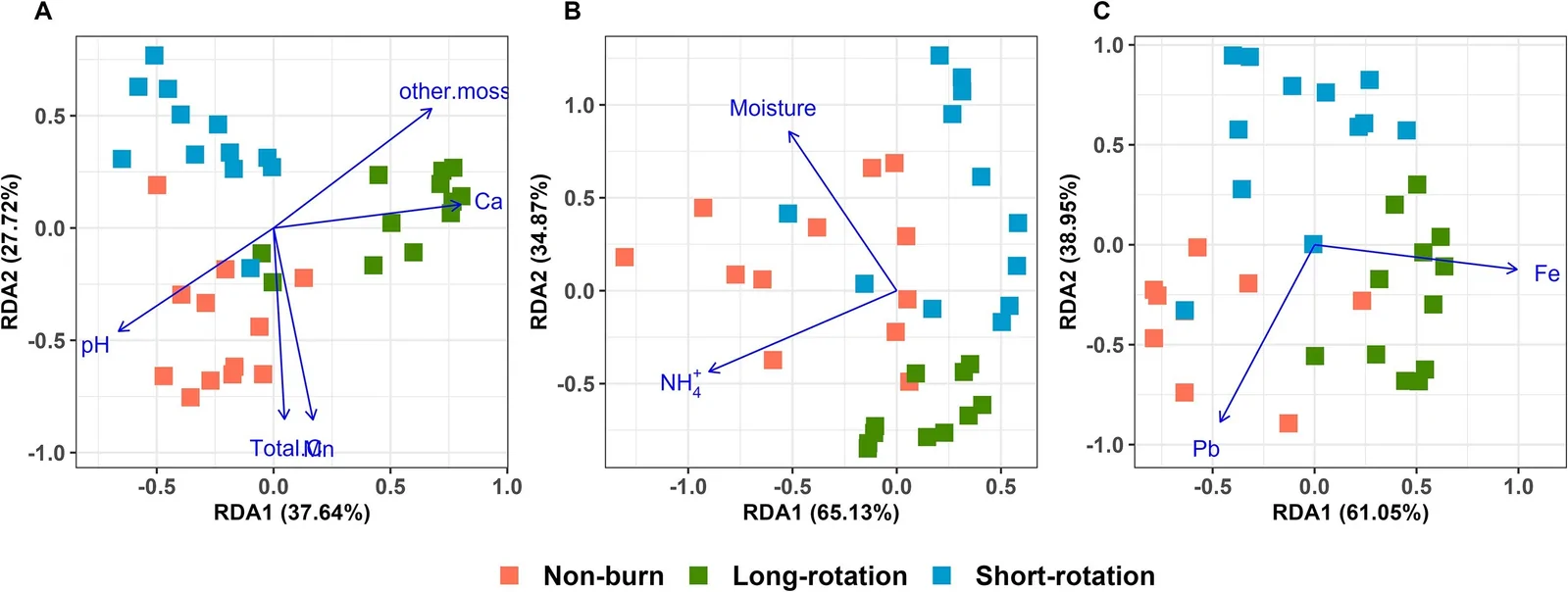
RDA ordination plots showing soil-related drivers of fungal communities for soil samples collected at three different depths under three prescribed burn regimes. Panels show A = 0–20 cm, B = 20–40 cm, C = 40–60 cm, under three prescribed burn regimes: Non-burn (unburned since 1954), long-rotation (20-year interval), and short-rotation (10-year interval). Only significant variables (*P* < 0.05) are shown. Sample sizes: Non-burn 0–20 cm (*n* = 12), non-burn 20–40 cm (*n* = 12), non-burn 40–60 cm (*n* = 9), long-rotation 0–20 cm (*n* = 12), long-rotation 20–40 cm (*n* = 11), long-rotation 40–60 cm (*n* = 12), short-rotation 0–20 cm (*n* = 12), short-rotation 20–40 cm (*n* = 12), short-rotation 40–60 cm (*n* = 12).

Forward selection redundancy analysis (RDA) was performed to investigate the relationships among environmental variables and fungal community structure. The most significant environmental factors that shaped fungal communities in the topsoil were Ca, Mn, % of other ‘non-*Sphagnum*’ moss cover, total C, and pH. NH_4_^+^ and moisture were the most important environmental factors in the 20–40 cm profile and Fe and Pb in the 40–60 cm profile. All final models were significant (ANOVA, *P* < 0.05) (Fig. 7).

### Functional guilds of fungal communities

**Figure 8:**
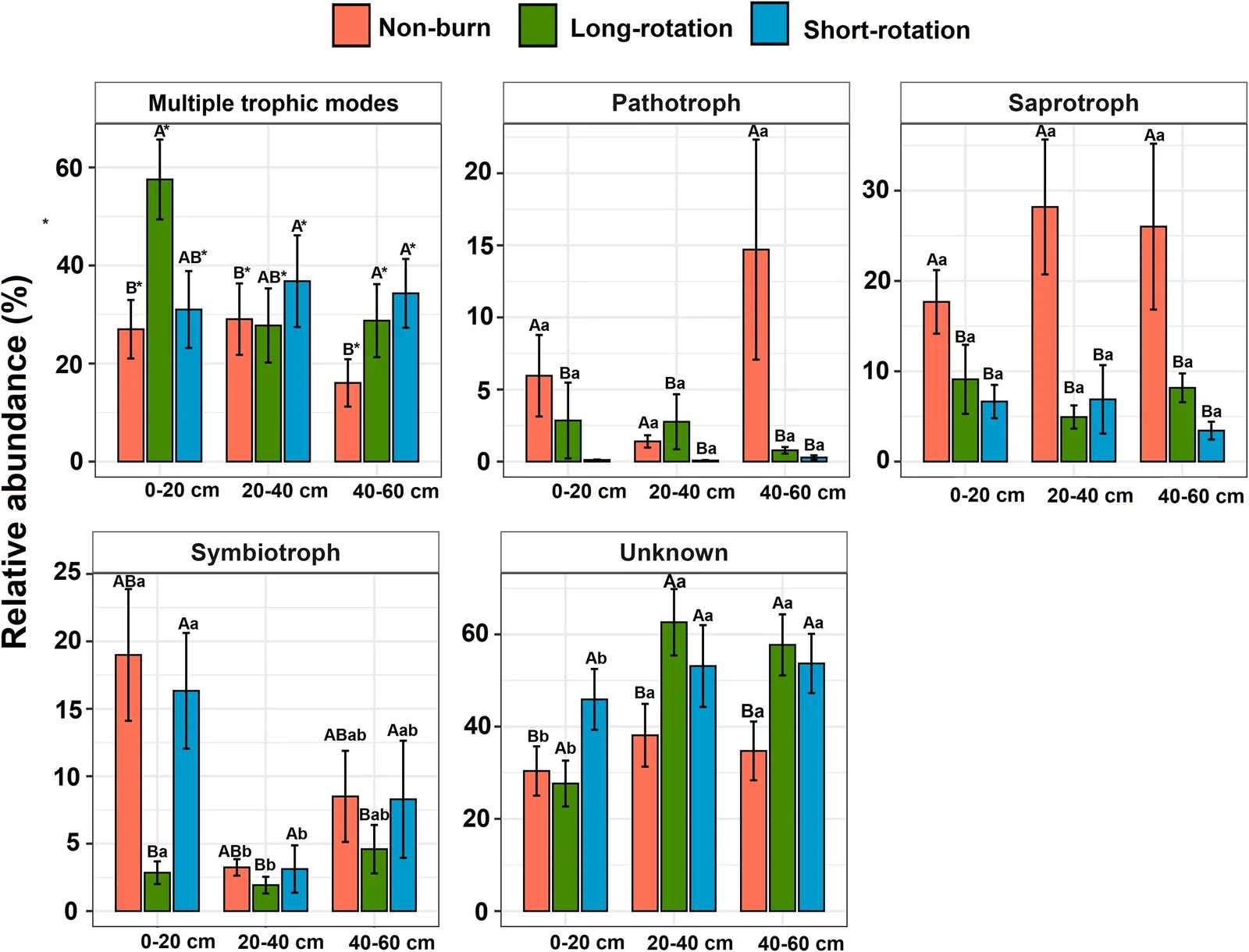
Relative abundance of fungal trophic modes assigned using FUNGuild across three soil depths under three prescribed burn regimes: Non-burn (unburned since 1954), long-rotation (20-year interval), and short-rotation (10-year interval). Bars represent mean values ± SE. Statistical differences were assessed using linear mixed-effects models applied to centred log-ratio (CLR) transformed data, with significance determined from Type III ANOVA and Tukey-adjusted pairwise comparisons (*P* < 0.05). Relative abundances are presented for ecological interpretation, while statistical significance is based on the CLR-transformed data. Uppercase letters indicate significant differences among burn treatments, and lowercase letters indicate significant differences among soil depths. Where the same lowercase letter is shared across all groups, no significant differences among soil depths were detected. Where a significant treatment × depth interaction was detected, letters indicate comparisons among burn treatments within each soil depth and are marked with an asterisk (*). Sample sizes: Non-burn 0–20 cm (*n* = 12), non-burn 20–40 cm (*n* = 12), non-burn 40–60 cm (*n* = 9), long-rotation 0–20 cm (*n* = 12), long-rotation 20–40 cm (*n* = 11), long-rotation 40–60 cm (*n* = 12), short-rotation 0–20 cm (*n* = 12), short-rotation 20–40 cm (*n* = 12), short-rotation 40–60 cm (*n* = 12).

FUNGuild classified 40% of the fungal ASVs according to their functional guild and trophic mode. Functional guild composition varied across burn regimes and soil depths (Fig. S4). The majority of classified fungi were assigned to the trophic mode of saprotrophs (17%), pathotrophs (6.5%), symbiotrophs (5%), and ASVs assigned to multiple trophic modes (11%) with the rest being classified ‘unknown’. Trophic modes differed among burn regimes as well as depth (Fig. 8). Pathotrophs were significantly affected by burn regime (*F*(2, 33.53) = 10.79, *P* < 0.001) but not depth (*F*(2, 65.16) = 0.20, *P* = 0.81). However, there was an increase in the non-burn subsoil (40–60 cm) where relative abundance increased to 14%. Saprotrophic fungi were affected by burn regime (*F*(2, 35.22) = 3.94, *P* = 0.04) but not depth (*F*(2, 64.34) = 2.11, *P* = 0.13) (Fig. 8; Table S5) and were more abundant in the non-burn control across all three depth profiles. Symbiotrophic fungi were affected by burn regime (*F*(2, 33.83) = 3.09, *P* = 0.04) as well as depth (*F*(2, 65.11) = 3.93, *P* = 0.02) with the relative abundance being highest in the topsoil of the non-burn control (18%). ASVs with multiple trophic modes showed a significant burn regime × soil depth interaction (*F*(4, 65.10) = 2.80, *P* = 0.03). Surface soils subject to long-rotation burning had the highest relative abundance of ASVs with multiple trophic modes (57%) (Fig. 8).

### Indicator species

Indicator analysis using the IndVal.g statistic identified a limited number of genera significantly associated with specific burn regime and depth combinations (*P* < 0.05; Table 1). In non-burn plots, *Entoloma* was identified as an indicator of the topsoil (0–20 cm), while four genera (*Gremmeniella, Neottiosporina, Collophora*, and *Sphaerulina*) were associated with the 40–60 cm depth. No indicators were detected in the 20–40 cm layer. In long-rotation plots, *Malassezia* and *Rhodotorula* were identified as unique indicators of the 40–60 cm depth, whereas no genera were uniquely associated with the topsoil or 20–40 cm layer. No single-indicator genera were detected in short-rotation plots. Analysis of multiple-group associations revealed several genera linked to burned treatments, particularly long-rotation and short-rotation plots (Table S6). These included *Devriesia*, which was associated with long-rotation topsoil and subsoil as well as short-rotation topsoil; *Solicoccozyma* and *Phialocephala*, which occurred across multiple long-rotation and short-rotation depth categories; and *Leucosporidium* and *Gymnopilus* which were associated with combinations of long-rotation and short-rotation subsoil layers. *Coniochaeta* was associated with long-rotation plots at 20–40 cm and 40–60 cm.

**Table 1:** Fungal genera identified as significant indicator taxa for individual burn regime × depth categories using indicator species analysis based on the IndVal.g statistic.

Genus	Statistic	*P* value	Indicator group
*Entoloma*	0.59	0.03	NB 0–20 cm
*Gremmeniella*	0.93	<0.001	NB 40–60 cm
*Neottiosporina*	0.57	0.003	NB 40–60 cm
*Collophora*	0.57	0.005	NB 40–60 cm
*Sphaerulina*	0.46	0.03	NB 40–60 cm
*Malassezia*	0.80	<0.001	LR 40–60 cm
*Rhodotorula*	0.61	<0.001	LR 40–60 cm

Only genera significantly associated with a single burn regime × depth category are shown. NB = Non-burn; LR = Long-rotation; SR = Short-rotation.

## Discussion

The observed shifts in fungal diversity and community composition across prescribed burn regimes and soil depths suggest that even low-intensity fire regimes can restructure belowground fungal assemblages, offering valuable insight into how prescribed burning influences soil fungal communities, which are critical to peatland ecosystem functioning.

### Soil fungal community characteristics

Community composition varied across burn regimes and soil depth. The most abundant phyla found in this study were Ascomycota and Basidiomycota (Fig. 1). Previous studies from peatland ecosystems have revealed the dominance and importance of these two groups, showing average relative abundances of 46% and 40%, respectively (Thormann and Rice 2007, Zhang et al. 2017, Juan-Ovejero et al. 2020). The large classes Leotiomycetes and Eurotiomycetes were found in this study and have been widely studied (Geiser et al. 2006, Asemaninejad et al. 2017, Ekanayaka et al. 2019). The class Leotiomycetes has important capabilities in lignocellulose degradation and root-associated fungi (Vrålstad et al. 2002), and is frequently observed in nutrient poor soils (Asemaninejad et al. 2017). Fire alters soil conditions, favouring fungal classes capable of colonizing post-fire environments and metabolizing carbon from fire residues such as charred wood or ash (Pulido-Chavez et al. 2023). The phylum Ascomycota is commonly associated with burning and has been known to increase in abundance after a fire (Robinson et al. 2008, Ammitzboll et al. 2021). Ascomycota was abundant across all treatments, including in the subsoil, suggesting that this group may act as a reservoir of propagules contributing to the recolonization of the topsoil following disturbance. However, it showed a slight decline in plots under a short-rotation regime (Fig. 1). Ascomycota have been known to dominate areas cleared for regeneration, becoming less dominant over time (Yan et al. 2018). In contrast, Basidiomycota increased in plots under a short-rotation regime, largely driven by Agaricomycetes, with many of these taxa assigned multiple trophic modes.

Indicator species analysis identified relatively few genera uniquely associated with individual burn regime and depth combinations, with most indicators occurring in non-burn and long-rotation treatments. In contrast, no genera were uniquely associated with short-rotation plots. This absence of single-indicator taxa in short-rotation plots may indicate reduced niche differentiation under the most frequently burned regime, with fungal communities instead dominated by more broadly distributed taxa. In long-rotation plots, indicator genera were primarily associated with deeper soil layers (40–60 cm), including Malassezia and Rhodotorula (Table 1). These taxa may reflect stress-tolerant communities persisting in deeper soil horizons, which are generally less directly affected by fire than the surface layers (Certini 2005). In contrast, non-burn plots supported a greater number of unique indicator taxa, particularly in the topsoil and deeper layers, consistent with greater niche partitioning under more stable environmental conditions. Analysis of taxa associated with multiple burn regime and depth combinations further supported this pattern. Several genera, including Devriesia, Solicoccozyma, and Phialocephala, were distributed across both long-rotation and short-rotation treatments and often spanned multiple soil depths (Table S6). Their widespread occurrence may indicate that repeated burning favours taxa with broader ecological tolerances or disturbance-tolerant life strategies, rather than strongly specialized or fire-exclusive taxa (Fox et al. 2022). Although Coniochaeta has been reported from fire-affected systems (Espinosa et al. 2023, Cuberos et al. 2024), its occurrence in this study was not restricted to a single burn regime or soil depth. This suggests that, in our system, prescribed burning did not strongly select for distinct pyrophilous indicator taxa, but instead favoured broadly distributed, disturbance-tolerant taxa.

### Impact of prescribed burning on fungal richness and community composition across soil profiles

We found that fungal richness was strongly affected by prescribed burning, with significant changes observed in the topsoil and persisting across the soil profile. Observed fungal richness was higher in non-burn control plots but significantly reduced under short-rotation burns, with a smaller decline observed in long-rotation burns (Fig. 3; Table S3). In long-rotation plots, this decline was accompanied by reduced relative abundance of genera such as Archaeorhizomyces and Serendipita, while Venturia was absent (Fig. S2), suggesting that some taxa may be less tolerant of repeated fire disturbance and associated environmental changes. As fungi play key roles in organic matter decomposition, nutrient cycling, and plant–fungal interactions, a reduction in fungal richness may affect these processes by reducing functional diversity and altering decomposition rates, nutrient availability, and plant–fungal associations (Juan-Ovejero et al. 2020).

Principal coordinate analysis showed that fungal community composition differed significantly among burn regimes and soil depths, although the proportion of explained variation was moderate (Fig. 4). These patterns mirror the effects of wildfire on soil in other ecosystems (Holden et al. 2016, Ammitzboll et al. 2021), highlighting the strong influence of burning regimes on fungal communities. The impact of prescribed burns on deeper soil horizons is particularly surprising, as peat soils are typically wet below 20 cm, which generally delays fire penetration. However, burning may indirectly affect these deeper layers by reducing plant root biomass (Tufekcioglu et al. 2010) and altering fungal communities closely linked to root systems. While changes in plant root biomass may partially explain the observed fungal shifts with depth, it is also important to consider that fungal communities can be influenced directly by fire-related disturbance and soil physicochemical changes. For example, fire can generate pyrogenic substrates that selectively favour certain saprotrophic or stress-tolerant taxa, independent of vegetation recovery (Fox et al. 2022). Additionally, fungal taxa show differential resilience to altered moisture, temperature, and oxygen regimes, particularly in stratified peat or organic soils (Semenova-Nelsen et al. 2019). Thus, the depth-related variation in fungal community composition likely reflects a combination of biotic and abiotic filtering processes, rather than vegetation change alone.

The observed decrease in fungal richness with depth (Fig. 3) can also be attributed to nutrient limitations and the anoxic conditions of peat soils (Koretsky et al. 2006). Furthermore, fungi generally recolonize more slowly than prokaryotes and are less resilient to land disturbance, with recovery processes mediated by both environmental conditions and plant physiological responses (Bardgett et al. 2013). For instance, burning may reduce the transfer of recently assimilated carbon from plants to fungi, limiting fungal recovery (Fuchslueger et al. 2014). As a result, the effects of burning on fungal communities, particularly in peatlands, may persist over a longer period of time (Zhou et al. 2019). These findings underscore the need for further research into the resilience of fungal taxa under different burning regimes, including immediate post-burn sampling to identify fire-tolerant taxa and their roles in ecosystem recovery.

### Community assembly processes

The results supported the second hypothesis that stochastic processes will dominate community assembly in burned plots. However, modified stochasticity ratio (MST) revealed depth-dependent differences in community assembly in the non-burn plots (Fig. 5). In non-burned plots, MST values indicated deterministic community assembly at 0–20 cm and 40–60 cm (MST < 50), suggesting that stable environmental conditions, resource heterogeneity, and strong environmental filtering dominate these soil layers (Fig. 5; Table S4). However, a shift to stochasticity in the 20–40 cm profile indicated weaker environmental selection at this intermediate depth potentially reflecting a zone with resource limitations, weaker environmental selection, or dispersal constraints that amplify ecological drift (Evans et al. 2017).

In contrast, both long-rotation and short-rotation plots exhibited MST > 50 at all depth profiles indicating that fungal community assembly processes are consistently dominated by stochastic mechanisms under prescribed burning (Fig. 5; Table S4). Macroecological theory suggests that increased resource supply can weaken environmental filtering and enhance stochastic assembly by reducing niche selection pressures (Chase 2010). Fire-induced disturbances likely disrupt environmental filters by altering soil structure and increasing resource heterogeneity (Philippot et al. 2021). Additionally, the frequent disturbance associated with rotation practices may prevent fungal communities from reaching equilibrium, weakening biotic interactions, and increasing the relative role of stochastic factors such as ecological drift and random colonization (Evans et al. 2017). Stochastic outcomes may be further influenced by fungal traits such as small spore size, high dispersal capacity, and rapid colonization, characteristics typical of disturbance-tolerant fungi. In contrast, fungi with slower growth rates or specialized symbiotic relationships may be filtered out under frequent burning due to reduced host availability and unstable conditions. While Chase (2010) emphasizes increased stochasticity under nutrient enrichment, our findings suggest that disturbance associated with prescribed burning may promote stochastic assembly processes, potentially through increased resource heterogeneity and disrupted environmental filtering.

Results from the Sloan community model showed poor fit (- *R^2^*) in most treatments with moderate fits only in non-burn plots at 0–20 cm and 20–40 cm (Fig. S3). The low *R^2^* values in burned plots and deeper soil layers suggest neutral processes such as ecological drift and dispersal limitation alone are insufficient to explain community assembly in these systems. The discrepancy between the NCM and MST results suggests that stochasticity detected in these systems is likely non-neutral. Fire-induced disturbances, resource heterogeneity, and spatially variable dispersal rates may amplify stochastic assembly processes that deviate from neutral assumptions. For example, patchy resource distributions in burned plots could drive stochastic outcomes tied to local conditions, while repeated prescribed burning over the long term may exacerbate priority effects and random colonization. Therefore, these results may arise because the MST captures both neutral and non-neutral stochasticity, whereas the NCM is limited to neutral assumptions (Burns et al. 2016). This is consistent with the presence of disturbance-tolerant genera such as *Coniochaeta*, whose rapid dispersal and thermotolerance enable them to dominate in fire-affected soils (Fox et al. 2022). These findings highlight the importance of incorporating both neutral and non-neutral processes when interpreting stochasticity in fungal community assembly.

Interestingly, niche width analysis revealed consistently higher niche breadth in non-burn plots across all depth profiles, suggesting that fungal taxa in these undisturbed soils are able to exploit a more diverse array of resources (Fig. 6B). This suggests that even in the 20–40 cm layer, where community assembly was driven by stochastic processes, taxa capable of utilizing a wide range of resources dominated the fungal community, maintaining functional diversity. Thus, stochastic assembly processes at this depth likely allowed for random colonization of different subsets of specialists, each adapted to specific or fragmented resource patches, thereby maintaining overall functional diversity. In contrast, the lower niche breadth observed in burned plots is likely a consequence of resource limitation and patchiness caused by burning, where fungal taxa rely on narrower and more specific niches (Carscadden et al. 2020).

### Environmental factors influencing fungal communities

RDA analysis showed that the most important factors driving fungal community composition were Ca, Mn, other moss cover, total C, and pH in the topsoil; NH_4_^+^ and moisture at 20–40 cm; and Fe and Pb at 40–60 cm (Fig. 7). Soil fungi are strongly influenced by edaphic factors (Vyas and Gupta 2014). The majority of studies investigating the impact of management regimes on fungal communities focus on SOM and pH, with less attention towards available cations. Importantly, changes in soil elements such as Ca, Mn, Fe, and Pb were among the main environmental factors shaping fungal communities. Several reviews have shown how fire affects soil physicochemistry (Augustine and McNaughton 1998, González-Pérez et al. 2004, Certini 2005, McSherry and Ritchie 2013, Zhou et al. 2017, Hrelja et al. 2020). Fungi are more sensitive to climatic conditions than archaea and bacteria, and they are known to follow similar environmental niche trends as plants (Bahram et al. 2018). Furthermore, many fungi are closely linked to plants through symbiotic associations and their dependence on plant-derived substrates such as litter, root exudates, and host metabolites (Antunes and Koyama 2017). For example, host metabolites are important for symbiotrophic fungi, whereas plant litter quality is an important determinant of saprotrophic fungal communities. Through these relationships, fungi influence key nutrient cycling processes such as decomposition, mineralization, and nutrient acquisition. While studies have demonstrated that fungal communities are major drivers of nutrient cycling and ecosystem multifunctionality (Schimel and Schaeffer 2012, Delgado-Baquerizo et al. 2016), the specific pathways linking fungal community shifts to nutrient dynamics remain context-dependent. In post-fire environments, altered soil chemistry, depth gradients, and the functional diversity of fungi introduce additional complexity to these relationships.

### Functional group shifts during prescribed burning

The relative abundance of saprotrophs was reduced in plots under long-rotation and short-rotation regimes, whereas the relative abundance of symbiotrophs was relatively high in the non-burn control and short-rotation regime. The ectomycorrhizal assignments included genera such as Tomentella, Laccaria, Amphinema, and Cortinarius, although the broader symbiotroph category also included non-ectomycorrhizal plant-associated taxa such as Serendipita, Phialocephala, and Sphaerulina. However, this pattern should be interpreted with caution, as the symbiotroph category includes a range of plant-associated fungi, including ericoid mycorrhizal taxa commonly associated with peatland vegetation, as well as taxa assigned to multiple trophic modes. In addition, FUNGuild assignments are based on curated functional information and may not fully capture the ecological complexity of all taxa, introducing some uncertainty into guild classification (Fig. 8; Table S5). It was expected that the relative abundance of saprotrophs would increase after fire as saprotrophs play a role in ecological succession and are part of an enrichment process after disturbance (Alem et al. 2020). Increased saprotroph abundance in the non-burn plots may reflect higher plant litter inputs, which serve as substrates for saprotrophic fungi (Boddy et al. 2007). Saprotrophs have been shown to play an important role in carbon cycling in peat bogs (Rice et al. 2006). While enzyme activity was not measured in this study, such shifts in the relative abundance of saprotrophic fungi could potentially translate to changes in decomposition dynamics. There was an increase in the abundance of fungi with multiple trophic modes in the topsoil of plots under a long-rotation regime. Similarly, increased abundances were observed in the 20–40 cm and 40–60 cm profiles of plots under a short-rotation regime. These findings may have implications for the distribution and persistence of functional guilds in peatlands under prescribed burning. Furthermore, the co-occurrence of these fungi has numerous benefits, such as exchanging water and nutrients via mycorrhizal hyphal networks (Brundrett 2002, 2004). Thus, these results provide insight into preserving functional guild diversity in peatlands under prescribed burning regimes.

## Conclusions

This study found that short-rotation prescribed burns significantly reduced fungal richness across all soil depths, with greater richness observed in non-burn plots, followed by long-rotation treatments. Stochastic processes dominated fungal community assembly in burned plots, likely due to increased resource patchiness and weakened environmental filtering. In contrast, deterministic processes were more prominent in non-burn plots, particularly in the surface (0–20 cm), and deeper (40–60 cm) layers, where more stable conditions support environmental filtering. Levins' niche width showed consistently broader niche breadth in non-burn plots, indicating the capacity of fungal taxa in undisturbed peatlands to exploit a wider array of soil resources.

These findings demonstrate that the impacts of prescribed burning extend beyond the intended modification of aboveground vegetation and into the soil microbial community, altering fungal guild composition and assembly mechanisms at least 60 cm deep. Such shifts have potential implications for peatland soil functioning, particularly in relation to organic matter decomposition and carbon storage. Understanding these belowground responses is vital for informing sustainable fire management practices and preserving the long-term ecological integrity of peatlands.

## Supplementary Material

fiag099_Supplemental_File
