# Supplementary material for: Effects of prescribed burning regimes on soil fungal communities, functional traits, and community assembly processes in surface and subsurface peat
Source: FEMS Microbiol Ecol. 2026 Sep 7;102(9):fiag099. doi: 10.1093/femsec/fiag099 (PMC13570591; doi:10.1093/femsec/fiag099)
Supplement: Supplementary file 1 [file fiag099_supplemental_file.docx]

**Supplementary information**

**Title: Effects of prescribed burning regimes on soil fungal communities, functional traits, and community assembly processes in surface and subsurface peat**

# Shaun M. Allingham^1,2,3^, Samantha J. Drake^2^, Andrew Ramsey^2,^ Chris D. Field^4^, Felix C. Nwaishi^5^, David R. Elliott^2^

^1^School of Biosciences, The University of Nottingham, Sutton Bonington, Loughborough, LE12 5RD, UK

^2^Nature-based Solutions Research Centre, University of Derby, Derby, DE22 1GB, UK

^3^NERC Environmental Omics Facility, School of Biosciences, University of Sheffield, Western Bank, Sheffield, S10 2TN, UK

^4^Department of Natural Sciences, Manchester Metropolitan University, Chester Street, Manchester, M1 5GD, UK

^5^Department of Earth and Environmental Sciences, Mount Royal University, 4825 Mt Royal Gate SW, Calgary, AB T3E 6K6, Canada

**Corresponding author.** Shaun M. Allingham. School of Biosciences, University of Nottingham, Nottingham, NG7 2RD, UK. Email: [shaun.allingham@nottingham.ac.uk](mailto:shaun.allingham@nottingham.ac.uk)

**Table S1**. Soil physicochemical properties across three different soil depths under three prescribed burn regimes. Data shows mean ± standard error (*n*=12).

| **Treatment and Depth (cm)** | **pH** | **Moisture (%)** | **Total N (%)** | **Total C (%)** | **NO3-** | **NH4+** | **Mg** | **Ca** | **Mn** | **Fe** | **Cu** | **Zn** | **P** | **Pb** | **K** | **Al** |
| --- | --- | --- | --- | --- | --- | --- | --- | --- | --- | --- | --- | --- | --- | --- | --- | --- |
| Non-burn 0-20 cm | 4.1 ± 0.06 | 78.22±0.38 | 1.26±0.02 | 50.50±1.06 | 2.88±0.46 | 16.88±1.62 | 916.4±215.08 | 1368.7±66.35 | 89.83±4.83 | 681.51±26.23 | 87.75±12.63 | 271.55±13.70 | 146.09±19.90 | 232.78±14.00 | 134.84±7.18 | 463.09±23.35 |
| Non-burn 20-40 cm | 4.21 ± 0.0.06 | 86.75±0.55 | 1.34±0.03 | 48.14±0.07 | 6.89±1.09 | 4.24±0.70 | 457.6±48.77 | 1842.5±95.47 | 52.67±4.16 | 830.03±79.28 | 14.75±1.03 | 117.25±2.67 | 138.3±4.88 | 39.79±2.54 | 86.43±9.17 | 431.56±30.53 |
| Non-burn 40-60 cm | 4.11 ± 0.0.7 | 89.27±0.38 | 1.41±0.04 | 46.74±0.38 | 4.04±0.36 | 3.04±0.44 | 414.76±32.73 | 1889.8±67.03 | 52.92±2.43 | 840.16±38.95 | 13.50±1.13 | 131.64±7.68 | 232.78±14.00 | 25.96±3.29 | 96.07±5.84 | 398.56±18.47 |
| Long-rotation 0-20 cm | 3.56±0.04 | 81.30±0.49 | 1.52±0.03 | 43.65±0.37 | 6.45±0.96 | 6.86±1.39 | 316.33±30.09 | 1782.00±54.32 | 67.89±4.58 | 1236.93±130.10 | 59.5±6.58 | 145.37±19.15 | 174.68±14.26 | 1.874±19.22 | 156.25±17.5 | 495.99±56.51 |
| Long-rotation 20-40 cm | 3.7±0.03 | 83.31±0.56 | 1.18±0.03 | 42.61±0.79 | 3.59±0.42 | 2.96±0.51 | 167.84±9.26 | 2194.5±136.34 | 47.92±4.50 | 1480.23±81.21 | 26.50±3.15 | 60.66±7.13 | 193.14±9.50 | 21.06±1.79 | 56.21±3.31 | 457.01±23.76 |
| Long-rotation 40-60 cm | 3.75±0.0.04 | 86.96±0.53 | 1.15±0.04 | 43.34±1.74 | 3.31±.0.24 | 2.69±0.24 | 163.69±6.88 | 2823.5±113.64 | 71.25±8.29 | 2079.39±103.41 | 17.50±1.94 | 63.27±7.20 | 1.874±19.22 | 13.56±2.35 | 78.54±7.78 | 561.57±33.22 |
| Short-rotation 0-20 cm | 3.81±0.0.02 | 82.89±0.79 | 1.36±0.09 | 37.87±1.72 | 3.51±0.24 | 4.05±1.29 | 211.46±14.22 | 1320.9±53.727 | 37.5±2.97 | 777.45±63.75 | 39.42±4.06 | 111.89±6.70 | 101.53±10.57 | 118.85±15.73 | 140.1±16.87 | 330.26±35.21 |
| Short-rotation 20-40 cm | 3.80±0.03 | 86.03±0.36 | 1.19±0.02 | 36.09±2.46 | 3.34±0.33 | 1.31±0.19 | 182.93±24.79 | 1754.6±116.72 | 24.83±1.33 | 824.47±68.569 | 15.00±2.72 | 44.06±3.33 | 144.16±4.37 | 11.49±1.49 | 40.68±4.57 | 249.53±25.26 |
| Short-rotation 40-60 cm | 3.86±0.0.03 | 88.41±0.71 | 1.01±0.02 | 46.11±0.32 | 3.18±0.25 | 1.19±0.17 | 167.69±21.00 | 2967.1±117.22 | 52.08±2.56 | 1395.4±56.09 | 15.17±1.05 | 31.38±4.21 | 118.85±15.73 | 13.27±1.07 | 67.48±11.33 | 355.58±31.67 |

**Fig. S1.**Rarefaction curves based on observed fungal species richness.

**Table S2.** Relative abundances (mean ± SE) of dominant soil fungal classes (>1%) across three soil depths under three prescribed burn regimes. **Relative abundances are presented for ecological interpretation**, while **statistical significance is based on Type III ANOVA from REML linear mixed-effects models applied to centered log-ratio (CLR) transformed data**. **Significant differences among groups are indicated by compact letter displays (CLD) derived from Tukey-adjusted pairwise comparisons (*P* < 0.05)**. Uppercase letters indicate differences among burn treatments and lowercase letters indicate differences among soil depths. Where the same lowercase letter is shared across all groups, no significant differences among soil depths were detected. Where a significant treatment × depth interaction was detected, letters indicate comparisons among burn treatments within each soil depth and are marked with an asterisk (*). ns = not significant.

| **Burn treatment** | **Depth (cm)** | Agaricomycetes | Archaeorhizomycetes | Dothideomycetes | Eurotiomycetes | Leotiomycetes | Sordariomycetes |
| --- | --- | --- | --- | --- | --- | --- | --- |
| **Non-burn** | **0-20 cm (*n*= 12)** | 0.23 ± 0.04 Aa | 0.10 ± 0.02 Aa | 0.07 ± 0.02 Aa | 0.10 ± 0.003 AB* | 0.38 ± 0.06 Bb | 0.03 ± 0.001 ns |
|  | **20-40 cm (*n*=12)** | 0.06 ± 0.01 Ab | 0.23 ± 0.08 Aa | 0.02 ± 0.04 Aa | 0.01 ± 0.002 B* | 0.53 ± 0.10 Bb | 0.01 ± 0.006 ns |
|  | **40-60 cm (*n*=9)** | 0.11 ± 0.04 Aab | 0.10 ± 0.06 Aa | 0.19 ± 0.10 Aa | 0.04 ± 0.02 A* | 0.39 ± 0.10 Bab | 0.008 ± 0.004 ns |
| **Long-rotation** | **0-20 cm (*n*=12)** | 0.04 ± 0.01 Ba | 0.06 ± 0.03Ba | 0.03 ± 0.02 Ba | 0.03 ± 0.01 A* | 0.68 ± 0.08 Bb | 0.007 ± 0.002 ns |
|  | **20-40 cm (*n*=11)** | 0.02 ± 0.006 Bb | 0.009 ± 0.01 Ba | 0.04 ± 0.02 Ba | 0.001 ± 0.009 B* | 0.76 ± 0.05 Bb | 0.02 ± 0.007 ns |
|  | **40-60 cm (*n*=12)** | 0.06 ± 0.02 Bab | 0.02 ± 0.003 Ba | 0.2 ± 0.008 Ba | 0.001 ± 0.006 B* | 0.71 ± 0.04 Bab | 0.02 ± 0.006 ns |
| **Short-rotation** | **0-20 cm (*n*=12)** | 0.29 ±0.06 Aa | 0.02 ± 0.003 Ba | 0.003 ± 0.001 Ba | 0.01± 0.007 A* | 0.46 ± 0.08 Ab | 0.06 ± 0.05 ns |
|  | **20-40 cm (*n*=12)** | 0.06 ± 0.02 Ab | 0.02 ± 0.002 Ba | 0.004 ± 0.002 Ba | 0.008 ± 0.004 AB* | 0.83 ± 0.05 Ab | 0.008 ± 0.004 ns |
|  | **40-60 cm (*n*=12)** | 0.13 ± 0.05 Aab | 0.01 ± 0.007 Ba | 0.009 ± 0.003 Ba | 0.005 ± 0.002 B * | 0.68 ± 0.08 Aab | 0.06 ± 0.003 ns |
| **Burn treatment** |  | ***F*(2, 95) = 26.21, *P* <0.001** | ***F*(2, 30.40) = 8.93, *P* < 0.001** | ***F*(2, 28.55) = 5.08, *P* = 0.01** | *F*(2, 34.25) = 3.03, *P* = 0.06 | ***F*(2, 33.80) = 6.29, *P* = 0.005** | *F*(2, 32.95) = 1.88, *P* = 0.16 |
| **Depth (cm)** |  | ***F*(2, 95) = 3.96, *P* = 0.02** | *F*(2, 64.25) = 1.77, *P* = 0.17 | *F*(2, 64.42) = 0.63, *P* = 0.535 | *F*(2, 64.88) = 0.98, *P* = 0.38 | ***F*(2, 64.35) = 4.80, *P* = 0.01** | *F*(2, 63.82) = 0.87, *P* = 0.42 |
| **Burn treatment** ×  **Depth** |  | *F*(4, 95) = 0.42, *P* = 0.79 | *F*(4, 64.09) = 0.22, *P* = 0.92 | *F*(4, 64.26) = 1.10, *P* = 0.36 | ***F*(4, 64.79) = 4.84, *P* = 0.002.** | *F*(4, 64.26) = 0.99, *P* = 0.42 | *F*(4, 63.72) = 1.67, *P* = 0.16 |

**Fig. S2.** Mean relative abundance (%) of the ten most abundant fungal genera across burn regimes (non-burn, long-rotation, and short-rotation) and soil depths (0-20 cm, 20-40 cm, and 40-60 cm). Point size and colour both represent relative abundance. Genera shown represent the most abundant taxa across all treatments.

**Table S3.** Fungal diversity indices across three soil depths under three prescribed burn regimes. Results are reported as **mean ± SE. Statistical significance (*P* < 0.05) is based on Type III ANOVA from REML linear mixed-effects models.** Significant differences among groups are indicated by **compact letter displays (CLD) derived from Tukey-adjusted pairwise comparisons of estimated marginal means.** Uppercase letters indicate differences among burn treatments and lowercase letters indicate differences among soil depths.

| **Burn treatment** | **Depth (cm)** | **Observed** | **Shannon** | **Simpson** |
| --- | --- | --- | --- | --- |
| **Non-burn** | **0-20 cm (*n*= 12)** | 60.16 ± 6.92 Aa | 2.56 ± 0.17 Aa | 0.83 ± 0.03 Aa |
|  | **20-40 cm (*n*=12)** | 39.08 ± 3.71 Ab | 2.13 ± 0.14 Ab | 0.75 ± 0.38 Ab |
|  | **40-60 cm (*n*=9)** | 53.2 ± 0.71 Aab | 2.31 ± 0.15 Aab | 0.80 ± 0.02 Aab |
| **Long-rotation** | **0-20 cm (*n*=12)** | 48.83 ± 5.49 Aa | 1.76 ± 0.15 Ba | 0.65 ± 0.04 Ba |
|  | **20-40 cm (*n*=11)** | 39.72 ± 4.63 Ab | 1.79 ± 0.16 Bb | 0.66 ± 0.05 Bb |
|  | **40-60 cm (*n*=12)** | 43.36 ± 3.64 Aab | 2.00 ± 0.15 Bab | 0.69 ± 0.04 Bab |
| **Short-rotation** | **0-20 cm (*n*=12)** | 36.66 ± 2.41 Ba | 1.90 ± 0.11 Ba | 0.72 ± 0.03 Ba |
|  | **20-40 cm (*n*=12)** | 27 ± 3.32 Bb | 1.20 ± 0.21 Bb | 0.48 ± 0.07 Bb |
|  | **40-60 cm (*n*=12)** | 33 ± 3.62 Bab | 1.65 ± 0.15 Bab | 0.63 ± 0.05 Bab |
| **Burn treatment** |  | ***F*(2, 33.35)** **= 12.04, *P* <0.001** | ***F*(2, 32.57)** **= 15.73, *P* <0.001** | ***F*(2, 32.67)** **= 9.58, *P*< 0.001** |
| **Depth (cm)** |  | ***F*(2, 64.61)** **= 7.59, *P*= 0.001** | ***F*(2, 64.35)** **= 4.38, *P* = 0.01** | ***F*(2, 64.70)** **= 4.26, *P* = 0.01** |
| **Burn treatment** × **Depth** |  | *F*(4, 64.50) = 0.61, *P* = 0.69 | *F*(4, 64.13) = 1.66, *P* = 0.17 | *F*(4, 64.52) = 1.89, *P* = 0.12 |

**Table S4.** Modified normalized stochasticity ratio (MST) of fungal communities across three different soil depths under three prescribed burn regimes.

| **Burn treatment** | **Depth (cm)** | **MST (%)** | **Deterministic processes** | **Stochastic processes** |
| --- | --- | --- | --- | --- |
| **Non-burn** | **0-20 cm (*n*= 12)** | 43 % | 62 % | 38 % |
|  | **20-40 cm (*n*=12)** | 71 % | 21 % | 78 % |
|  | **40-60 cm (*n*=9)** | 32 % | 72 % | 28 % |
| **Long-rotation** | **0-20 cm (*n*=12)** | 59 % | 31 % | 69 % |
|  | **20-40 cm (*n*=11)** | 69 % | 27 % | 73 % |
|  | **40-60 cm (*n*=12)** | 70 % | 21 % | 78 % |
| **Short-rotation** | **0-20 cm (*n*=12)** | 56 % | 43 % | 57 % |
|  | **20-40 cm (*n*=12)** | 60 % | 40 % | 60 % |
|  | **40-60 cm (*n*=12)** | 62 % | 34 % | 66 % |

**Fig. S3.** Estimation of the neutral processes based on Sloan’s neutral model. The parameters m (migration rate) and *R^2^* (model fit) represents the migration rate and goodness of fit respectively. ASVs occurring more or less frequently than predicted are shown in blue and red dots respectively. Dashed lines represent 95% confidence intervals and the species falling within the confidence intervals are considered to be neutrally distributed. **(A)** Non-burn 0-20 cm, **(B)** Non-burn 20-40 cm, **(C)** Non-burn 40-60 cm, **(D)** Long-rotation 0-20 cm, **(E)** Long-rotation 20-40 cm, **(F)** Long-rotation 40-60 cm, **(G)** Short-rotation 0-20 cm, **(H)** Short-rotation 20-40 cm, **(I)** Short-rotation 40-60 cm.

**Table S5.** Relative abundances (mean ± SE) of trophic modes derived from funGUILD across three different soil depths under three prescribed burn regimes. **Relative abundances are presented for ecological interpretation**, while **statistical significance is based on Type III ANOVA from REML linear mixed-effects models applied to centered log-ratio (CLR) transformed data. Significant differences among groups are indicated by compact letter displays (CLD) derived from Tukey-adjusted pairwise comparisons (*P* < 0.05)**. U**ppercase letters indicate differences among burn treatments and lowercase letters indicate differences among soil depths**. Where the same lowercase letter is shared across all groups, no significant differences among soil depths were detected. Where a significant treatment × depth interaction was detected, letters indicate comparisons among burn treatments within each soil depth and are marked with an asterisk (*).

| **Burn treatment** | **Depth (cm)** | **Pathotroph** | **Saprotroph** | **Symbiotroph** | **Multiple**  **trophic modes** |
| --- | --- | --- | --- | --- | --- |
| **Non-burn** | **0-20 cm (*n*= 12)** | 0.05 ± 0.02 Aa | 0.17 ± 0.03 Aa | 0.18 ± 0.04 ABa | 0.26 ± 0.05 B* |
|  | **20-40 cm (*n*=12)** | 0.01 ± 0.0001 Aa | 0.28 ± 0.07 Aa | 0.03 ± 0.007 ABb | 0.29 ± 0.07 B* |
|  | **40-60 cm (*n*=9)** | 0.14 ± 0.007 Aa | 0.26 ± 0.08 Aa | 0.08 ± 0.003 ABab | 0.16 ± 0.04 B* |
| **Long-rotation** | **0-20 cm (*n*=12)** | 0.02 ± 0.0002 Ba | 0.09± 0.03 Ba | 0.02 ± 0.008 Ba | 0.57 ± 0.08 A* |
|  | **20-40 cm (*n*=11)** | 0.02 ± 0.0002 Ba | 0.049 ± 0.01 Ba | 0.01 ± 0.006 Bb | 0.27 ± 0.07 AB* |
|  | **40-60 cm (*n*=12)** | 0.0007 ± 0.0001 Ba | 0.06 ± 0.01 Ba | 0.04 ± 0.001 Bab | 0.28 ± 0.07 B* |
| **Short-rotation** | **0-20 cm (*n*=12)** | 0.001 ±0.0002 Ba | 0.16 ± 0.003 Ba | 0.16 ± 0.004 Aa | 0.31 ± 0.07 AB* |
|  | **20-40 cm (*n*=12)** | 0.0007 ± 0.0003 Ba | 0.03 ± 0.01 Ba | 0.03 ± 0.01 Ab | 0.36 ± 0.009 A* |
|  | **40-60 cm (*n*=12)** | 0.002 ±0.001 Ba | 0.08 ± 0.04 Ba | 0.08 ± 0.04 Aab | 0.34 ± 0.07 A* |
| **Burn treatment** |  | ***F*(2, 33.53) = 10.79, *P* < 0.001** | ***F*(2, 35.22) = 3.94, *P* = 0.04** | ***F*(2, 33.83) = 3.09, *P* = 0.04** | ***F*(2, 33.44) = 12.33, *P* < 0.001** |
| **Depth (cm)** |  | *F*(2, 65.16) = 0.20, *P* = 0.81 | *F*(2, 64.34) = 2.11, *P* = 0.13 | ***F*(2, 65.11) = 3.93, *P* = 0.02** | *F*(2, 65.15) = 0.45, *P* = 0.63 |
| **Burn treatment**  × **Depth** |  | *F*(4, 65.11) = 1.90, *P* = 0.12 | *F*(4, 64.27) = 1.62, *P* = 0.18 | *F*(4, 96) = 0.45, *P* = 0.76 | ***F*(4, 65.10) = 2.80, *P* = 0.03** |

**Fig. S4.** Relative abundance of functional guilds across three different soil depths under three prescribed burn regimes: Non-burn (unburned since 1954), long-rotation (20-year interval), and short-rotation (10-year interval). Sample sizes: Non-burn 0-20 cm (*n* = 12), non-burn 20-40 cm (*n* = 12), non-burn 40-60 cm (*n* = 9), long-rotation 0-20 cm (*n* = 12), long-rotation 20-40 cm (*n* = 11), long-rotation 40-60 cm (*n* = 12), short-rotation 0-20 cm (*n* = 12), short-rotation 20-40 cm (*n* = 12), short-rotation 40-60 cm (*n* = 12).

**Table S6**. Fungal genera significantly associated with multiple burn regime × depth combinations, identified using indicator species analysis based on the IndVal.g statistic. These taxa represent broadly distributed or generalist responses across treatments. Indicator groups represent all burn regime × depth categories with which each genus showed a significant association (*P* < 0.05). NB = Non-burn; LR = Long-rotation; SR = Short-rotation.

| **Genus** | **Statistic** | **p value** | **Indicator Group** |
| --- | --- | --- | --- |
| *Cladophialophora* | 0.89 | <0.001 | NB 0-20cm; NB 40-60cm |
| *Devriesia* | 0.70 | <0.001 | LR 0-20cm; LR 40-60cm; SR 0-20cm |
| *Penidiella* | 0.83 | <0.001 | NB 0-20cm; NB 40-60cm |
| *Protoventuria* | 0.79 | <0.001 | NB 0-20cm; NB 40-60cm |
| *Venturia* | 0.83 | <0.001 | NB 0-20cm; NB 40-60cm |
| *Solicoccozyma* | 0.69 | <0.001 | LR 0-20cm; LR 20-40cm; LR 40-60cm; SR 0-20cm; SR 40-60cm |
| *Phialocephala* | 0.65 | 0.001 | LR 0-20cm; LR 20-40cm; LR 40-60cm; SR 0-20cm; SR 20-40cm; SR 40-60cm |
| *Penicillium* | 0.53 | 0.008 | LR 0-20cm; LR 40-60cm; NB 40-60cm; SR 40-60cm |
| *Pezoloma* | 0.85 | 0.008 | LR 0-20cm; LR 40-60cm; NB 0-20cm; NB 20-40cm; NB 40-60cm; SR 0-20cm; SR 20-40cm; SR 40-60cm |
| *Mycena* | 0.58 | 0.009 | LR 0-20cm; LR 40-60cm |
| *Coniochaeta* | 0.53 | 0.01 | LR 20-40cm; LR 40-60cm |
| *Piskurozyma* | 0.52 | 0.01 | LR 20-40cm; NB 40-60cm |
| *Teratosphaeria* | 0.73 | 0.03 | LR 0-20cm; LR 20-40cm; LR 40-60cm; NB 0-20cm; NB 20-40cm; NB 40-60cm |
| *Leucosporidium* | 0.47 | 0.04 | LR 20-40cm; LR 40-60cm; SR 40-60cm |
| *Gymnopilus* | 0.62 | 0.04 | LR 40-60cm; SR 0-20cm; SR 40-60cm |
